# Clinical scale of artificial intelligence-based nerve segmentation on ultrasound: a pilot validation study

**DOI:** 10.3389/fmed.2026.1879747

**Published:** 2026-08-20

**Authors:** Bernard V. Delvaux, Yoann Elmaleh, Karim Guessous, Thierry Garnier, Bertrand Morel, Killian Yao, Thomas Giral, James S. Bowness, Sandrine Herbelet

**Affiliations:** 1 Ramsay Santé, Claude Galien Private Hospital, Quincy-Sous-Sénart, France; 2 Department of Anaesthesia, University College London Hospitals NHS Foundation Trust, London, United Kingdom; 3 Department of Targeted Intervention, University College London, London, United Kingdom; 4 Department of Epidemiology and Data Science, Amsterdam UMC, Vrije Universiteit Amsterdam, Amsterdam, Netherlands

**Keywords:** artificial intelligence, evaluation, medical devices, regional anesthesia, scale, translational AI, ultrasound, validation

## Abstract

**Background:**

The objective metrics for AI-based nerve segmentation are time-consuming and may not fully reflect their clinical usefulness. We aimed to 1) conduct a pilot validation study of the Clinical Segmentation Evaluation Scale (C-SES), as an estimation for an objective metric, and 2) establish a threshold on those scales, because the thresholds were derived for both the new C-SES scale AND for objective metrics (IoU, DSC) to determine the usefulness of AI predictions.

**Methods:**

Seven experts rated 74 ultrasound sequences twice using AI-based nerve recognition software (cNerve) in 3 anatomical regions. In Step 1, the C-SES was evaluated for 1) content validity (expert appraisal of relevance, comprehensiveness, and clarity), 2) concurrent criterion validity (by correlating the C-SES with a reference standard, the Intersection over Union—IoU), and 3) reliability (intra and inter-rater) and error measurement. In an additional Step 2, expert-perceived usefulness for non-experts and novice anesthesiologists was evaluated to derive candidate thresholds for Intersection over Union (IoU) and C-SES metrics within the investigated clinical context.

**Results:**

In Step 1, content validity was preliminarily supported by expert consensus. Concurrent criterion validity was strong (Pearson’s *r* = 0.861). Reliability was excellent within raters [mean ICC(2,1) = 0.905] and high for aggregated inter-rater ratings [ICC(2,3) = 0.869; ICC(2,k) = 0.940]. Precision increased when several raters were pooled. In Step 2, the Youden thresholds for non-expert usefulness were 0.206 for the IoU and 3.51 for the C-SES; for novice usefulness, the thresholds were 0.299 and 4.689, respectively.

**Conclusion:**

In this pilot validation, the C-SES appeared to estimate objective segmentation metrics, although further validation is needed. The proposed thresholds are preliminary and hypothesis-generating rather than universal clinical benchmarks and require confirmation before generalization beyond expert assessors and this spectrum-enriched sample.

## Introduction

Artificial intelligence (AI) is widely available to assist with anatomy recognition in various imaging conditions. Among the different approaches used to enhance anatomy visualization, semantic segmentation—defined as the precise delineation of a region of interest—is the method commonly implemented.

**Figure 1:**
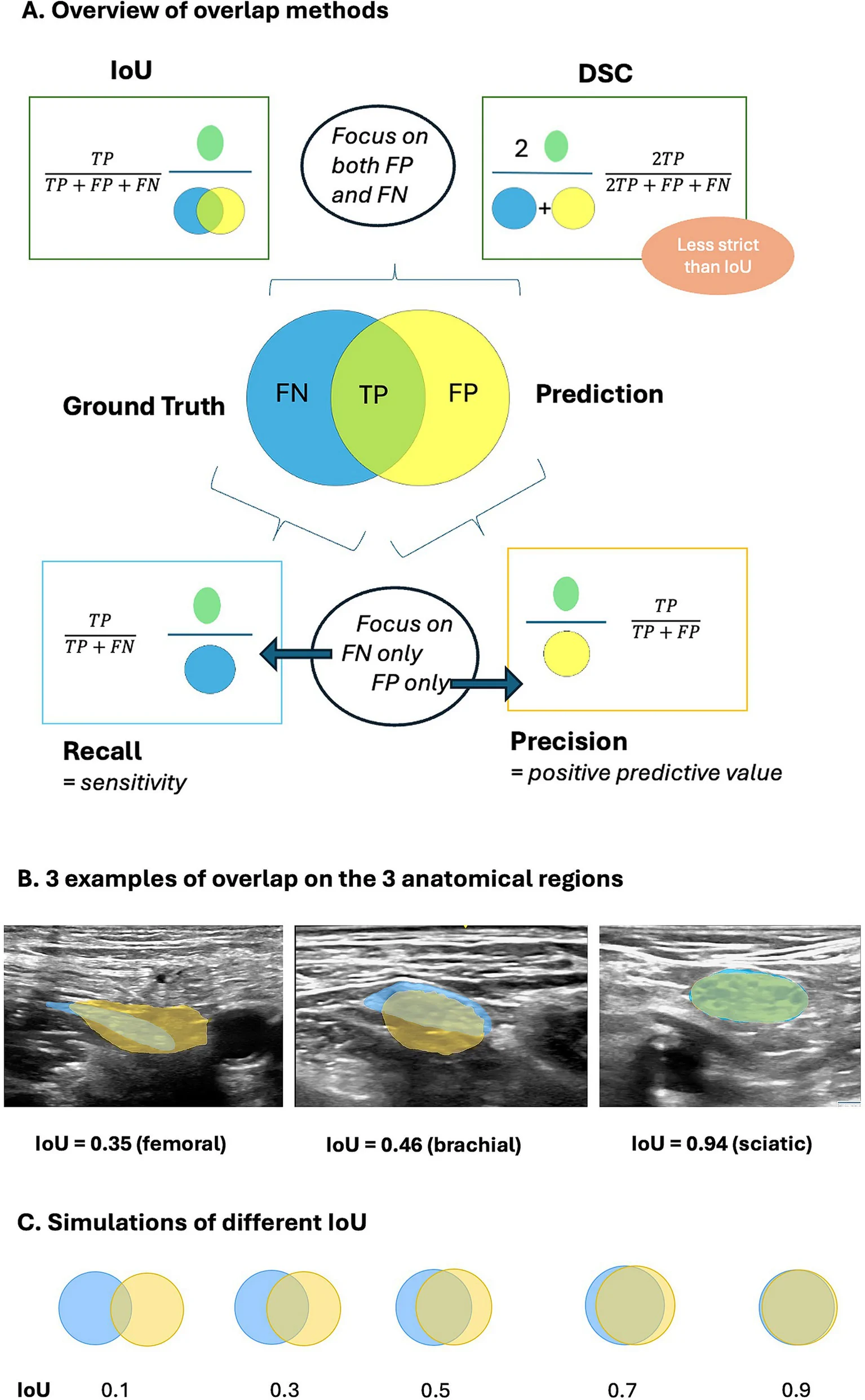
Overlap methods. Left **(A)** Overview of the main overlap metrics. Top right **(B)** Examples of overlap and its corresponding IoU on the three studied anatomical regions (femoral, brachial, sciatic). Bottom right **(C)** Graphical simulation of different IoU values. IoU, Intersection over union; DSC, Dice similarity coefficient; FP, False positive; TP, True positive; FN, False negative.

The performance of these segmentation models is usually assessed by comparing the overlap between a prediction and an expert-defined annotation used as a reference standard. This allows calculating standard overlap metrics, particularly Intersection over Union (IoU) and the Dice Similarity Coefficient (DSC) (1–3). These metrics provide a global estimate of segmentation quality by combining the two principal sources of error, false-positive and false-negative classifications, into a single performance measure. They are summarized in Figure 1.

However, the annotation process required by this approach is highly time-consuming. As a result, the possibility of replacing metric-based assessment with expert judgment has been raised in the literature (4, 5). This approach has been reported in the literature (2, 4, 6–8) but not yet formally validated. In this study, we use AI-based nerve recognition on ultrasound (9, 10) as a use case to perform preliminary validation of a single-item expert scale of segmentation performance, using a standard validation methodology.

## Methods

Details of the methodology are reported in Appendix S1. This study was approved by a national ethics committee (IRB number IRB00010835).

### Construct and reporting

This study is based on the consensus-based Standards for the selection of health Measurement INstruments (COSMIN) methodology (11–13) adapted to clinician-reported outcome measures. In keeping with this framework, content validity, construct validity (hypothesis testing), criterion validity, and intra and inter-rater reliability are assessed. These steps are outlined in Table 1.

**Table 1:** Main steps of the COSMIN-based validation framework used in this study.

COSMIN domain	Explanation of the concept	Typical test and expected result	Application in this study
Content validity	The item should be relevant, comprehensive, and understandable for the construct it is intended to measure.	Qualitative appraisal based on expert judgment, literature, and conceptual analysis. Ideally, it should be done formally by a focus group such as Delphi.	A single 0–10 item, adapted from Bowness *et al.*, was used to rate overall AI-based nerve highlighting performance. Content validity was appraised with respect to the perceived overlap between AI highlighting and the nerve region of interest, including relevance, comprehensiveness, and comprehensibility for expert raters in ultrasound-guided regional anesthesia.
Criterion validity—concurrent validity	The score should show a strong association with a reference standard (when this reference standard exists).	Typically assessed using correlation and/or agreement with the reference standard. A strong positive association is expected.	Ratings from 7 experts assessed at 2 time points were aggregated using a linear mixed-effects model to derive one latent sequence-level score (seg_latent). Concurrent validity was then examined against IoU (reference standard) with Pearson’s *r* and Spearman’s *rho*. Linear mixed regression was additionally fitted to quantify the magnitude of association.
Hypothesis testing for construct validity	The score should show some degree of association with a related construct, according to pre-specified hypotheses regarding direction and magnitude of association	Typically assessed using correlation and/or agreement with the other measures. A moderate to strong positive/negative association is expected according to the chosen measure.	The association of seg_latent with precision and recall was examined. Moderate to strong positive correlation was expected, as precision and recall capture only part of the IoU (convergent validity)
Reliability—intra-rater	The same rater should provide similar scores when reassessing the same cases.	Typically assessed using intraclass correlation coefficients (ICCs) or kappa coefficient. A time interval of at least 2 weeks is required. High agreement is expected.	7 expert anesthesiologists rated all 74 sequences twice, with a 3-week interval. Intra-rater reliability was assessed separately for each expert and then summarised across raters using mean (SD) and median (IQR) of the ICC estimates.
Reliability—inter-rater	Different raters should provide similar scores when evaluating the same cases.	Typically assessed using intraclass correlation coefficients (ICCs) or kappa coefficient. High agreement is expected	Inter-rater reliability was assessed across the 7 experts separately at the first and second rating round, and after averaging both rounds. Because the intended use of the scale was to support generalisation beyond the specific raters included, a two-way random-effects absolute-agreement ICC framework was prespecified, and ICC (2,1), ICC (2,3), and ICC(2,k) were reported.
Measurement error	Quantifies the absolute amount of error in the score, expressed in the unit of the scale. Unlike ICC, which assesses relative reliability, measurement error indicates how much an individual score may vary on repeated assessment.	Typically assessed using standard error of measurement (SEM), smallest detectable change (SDC), and Bland–Altman Limits of agreement (LoA). Low values are expected.	Measurement error was assessed using the test–retest ratings. For each rater, differences between the second and first rating rounds were calculated. SEM, individual SDC, and Bland–Altman LoA were estimated per rater and in a pooled analysis across all complete patient–rater pairs. Results were expressed in C-SES points.

### Study design and data source

This methodological validation study used retrospective ultrasound video sequences acquired in accordance with GRAITE-USRA guidelines (14) on a Venue ultrasound system (GE HealthCare, Chicago, United States) with AI-based nerve recognition software (cNerve, R4 version), which highlights nerves from three anatomical regions: the brachial plexus at the interscalene region, the femoral nerve at the inguinal region, and the sciatic nerve at the popliteal fossa. A spectrum-enriched validation sample was used, selecting sequences 1) to cover a broad range of segmentation quality for each anatomical region, with approximately balanced poor, intermediate, and good cases (20, 30, and 24 of the 74 sequences, respectively; per-region counts in Appendix S1, Table 1) and 2) to have visible nerve structure in the whole sequence. Hence, performance in this sample did not reflect performance in an unselected clinical population.Reference annotation and metrics

The reference annotation was obtained by a three-expert panel, using predefined segmentation rules. Objective overlap metrics recommended for overlap measurement (IoU, DSC, precision, recall) were calculated at the sequence level from frames extracted every 50 ms. Three of the seven C-SES raters also served on this reference-annotation panel, whereas the remaining four raters had no role in generating the reference annotation.

**Table 2:** Step 1—main results (validation of a subjective segmentation scale).

Domain	Variable	Analysis	Estimate [95% CI]	Interpretation
Latent score derivation	seg_latent	Mixed model intercept	5.017	Between-sequence variability is the largest structured source of variance, while the rater effect is smaller and the test–retest component is limited
		SD(patient)	2.104	
		SD(evaluator)	0.471	
		SD (evaluator:retest)	0.183	
		Residual SD	1.374	
Concurrent validity	IoU—seg_latent	Pearson’s r	0.861*** [0.787; 0.910]	Very strong correlation
		Spearman rho	0.866*** [0.804; 0.904]	
		Linear regression slope *β*	0.075*** [0.065; 0.085] *R*^2^ = 0.741; RSE = 0.093	*seg_latent* is a strong predictor of IoU—the model explains 74.1% of the variance in IoU
Hypotheses testing for Construct validity	Precision—seg_latent	Pearson’s r	0.498*** [0.305; 0.653]	Moderate correlation
	Recall—seg_latent	Pearson’s r	0.842*** [0.759; 0.897]	Very strong correlation
Reliability	C-SES	Intra-rater ICC (2,1), mean	0.905 SD 0.032	Good (0.899) to excellent (0.905) intra-rater agreement
		Intra-rater ICC (2,1), median	0.899 IQR 0.035	
		Inter-rater ICC (2,1), 2 passes	0.689 [0.602; 0.770]	Moderate agreement for a single rater—good to excellent agreement for several raters
		Inter-rater ICC (2,3), 2 passes	0.869 [0.820; 0.909]	
		Inter-rater ICC(2,k), 2 passes	0.94 [0.914; 0.959]	
Measurement error	C-SES (single rater)	Test–retest bias, mean	−0.26 T1-T2 pooled analysis	Scores were slightly lower at the second rating round, but the average bias was small on a 0–10 scale.
		SEM	0.76 C-SES-points	A single C-SES rating has a typical test–retest measurement error of approximately 0.8 points.
		Individual SDC	2.12 C-SES-points	For an individual rating, a change smaller than approximately 2 points may fall within expected measurement error.
		Bland–Altman LoA	−2.38 to +1.86	For 95% of repeated ratings, the second rating is expected to lie approximately 2.4 points lower to 1.9 points higher than the first rating.
	C-SES (estimation for 3 raters)	Test–retest bias, mean	−0.26 (SD 0.11)	
		SEM for 3-rater mean	0.43 (0.05)	The test–retest measurement error decreases from 0.8 to 0.43 points.
		SDC for 3-rater mean	1.18 (0.13)	For an individual rating, a change smaller than approximately 1 point now may fall within expected measurement error.
		Bland–Altman LoA	−1.44 to 0.92	For 95% of repeated ratings, the second rating is expected to lie approximately 1.44 points lower to 0.92 points higher than the first rating

CI, Confidence interval; SD, Standard deviation; IQR, Interquartile range; IoU, Intersection over union; ICC, Intraclass correlation coefficient; RSE, Residual standard error; ***: *p* value < 0.001.

### Expert rating procedure

Seven experts with at least 5 years of experience in regional anesthesia, who were blinded to the reference annotation, independently rated all AI-annotated video sequences on two occasions with an interval of 3 weeks. They used the Clinical Segmentation Evaluation Scale (C-SES), the instrument under validation, that is a single 0–10 item adapted from Bowness et al. (4). Written instructions specified that raters should rate perceived overlap between AI highlighting and the nerve region—from 0 (very poor) to 10 (excellent)—considering only false-positive and false-negative highlighting and should not score anything else (such as clinical usefulness).

Two additional binary items (yes-no)—also adapted from Bowness et al. (4)—assessed whether the AI-based highlighting can help guide the procedure for a non-expert anesthetist (*usefulness*) and for a novice (*usefulness_novice*) (Appendix S1). As these items capture expert judgments about hypothetical non-expert and novice users rather than outcomes measured in actual users, they are referred to as expert-perceived usefulness. A non-expert is referred to as a qualified anesthesiologist who does not perform regional anesthesia on a daily basis but has performed the 16–20 blocks per anatomical region required to meet the minimum proficiency standards (15).

### Validation framework

The primary objective (step 1) was to evaluate the measurement properties of this single-item C-SES with respect to the COSMIN framework. The secondary objective (step 2) was to use the two binary usefulness items to derive thresholds based on expert-perceived usefulness for the IoU and the C-SES.

### Step 1: validation of the single-item C-SES

#### Preliminary content validity

Initial content validity was established qualitatively through a preliminary expert consensus process using the COSMIN domains of 1) relevance (targeting the perceived overlap between AI highlighting and the nerve region of interest), 2) comprehensiveness (the item captured overall overlap performances rather than a single subcomponent of performance), and 3) comprehensibility (based on phrasing in simple terms) by all anesthesia experts (Appendix S3).

#### Concurrent criterion validity

As a preliminary step, the repeated subjective segmentation ratings were aggregated into a single sequence-level score to enable comparison with the objective reference metric (IoU). To achieve this, we used a *linear mixed-effects model* separating the variability attributable to the sequence itself from that related to differences between raters and test–retest variation (Appendix S1). As a sensitivity analysis, the latent segmentation score (*seg_latent*) was calculated by the arithmetic mean of all 14 raw ratings per sequence. The mixed-effects model comprised a fixed intercept and crossed random intercepts for sequence, rater, and rating round (nested within rater) and was estimated by restricted maximum likelihood (REML, lme4); each sequence-level score (seg_latent) was obtained as the fixed intercept plus the sequence random-effect conditional mode (empirical Bayes/BLUP).

Concurrent validity was then evaluated by Pearson’s and Spearman’s correlations between seg_latent and IoU. A strong to very strong association was expected. To quantify the relationship between the two variables, a linear regression model was fitted with IoU as the dependent variable and *seg_latent* as the predictor.

#### Construct validity

Additionally, convergent validity by hypothesis testing for construct validity evaluated the correlation between seg_latent and the 2 components of the IoU, precision and recall, expecting a moderate to strong correlation.

#### Reliability

Reliability testing expected high intra and inter-rater agreements (Appendix S1). Intra-rater reliability was assessed by comparing the first and second ratings of each expert across all sequences. For each expert, a two-way random-effects intraclass correlation coefficient (ICC) for absolute agreement was calculated (16), and the seven coefficients were then summarised using the mean and standard deviation (SD) and the median and interquartile range (IQR).

Inter-rater reliability was assessed at each rating round and on the within-rater mean of the two rounds. Because the intended use of the scale was to support generalization beyond the specific raters included in the study, a two-way random-effects absolute-agreement model (ICC) was prespecified. Inter-rater results were reported as ICC (2,1), ICC(2,k), where k=7 , and ICC (2,3) as an additional clinically relevant estimation corresponding to the average of three raters (Appendix S1).

#### Measurement error

Measurement error quantifies the absolute amount of error in the score, expressed in the unit of the scale. Standard error of measurement (SEM), individual smallest detectable change (SDC), and Bland–Altman limits of agreement (LoA) were calculated separately for each rater and in a pooled analysis including all complete patient–rater pairs (16). To reflect a potential multi-rater use of the C-SES, measurement error was additionally estimated for three-rater (Appendix S1).

### Step 2: derivation of thresholds based on expert-perceived usefulness

#### Aggregation of binary usefulness judgments

As a preliminary step, the usefulness evaluations of all experts were aggregated into a single sequence-level score (Appendix S1). A sequence was considered clinically useful by consensus when 60% of the ratings were positive (*usefulness_prop* and *usefulness_novice_prop > 0.6*). Sensitivity analyses repeated the threshold derivation using alternative dichotomization cut-offs of 0.5, 0.7, 0.8 and 0.9. Aggregation of usefulness was also performed by a mixed logistic model as sensitivity analyses (Appendix S1).

#### Reliability and construct validity of the binary usefulness items

Before threshold derivation, the reliability and construct validity of the binary usefulness items were assessed separately for *usefulness* and *usefulness_novice* (Appendix S1). Intra-rater reliability was evaluated using Cohen’s unweighted kappa. Inter-rater reproducibility was assessed using an exact multi-rater kappa (Conger’s extension). As an additional hypothesis-testing construct validity analysis, Pearson’s correlation was calculated between the aggregated usefulness values (*usefulness_prop* and *usefulness_novice_prop*) and IoU, with an expected moderate to strong positive association. % positive and negative agreement was also calculated.

#### ROC-based threshold derivation

Receiver operating characteristic (ROC) analyses were then performed for both novices and non-experts to identify candidate thresholds for IoU and for *seg_latent* against each binary clinical reference standard. The primary threshold selection criterion was the Youden index, as sensitivity and specificity were considered of comparable clinical importance. Two secondary operating rules were also reported: the threshold yielding the highest specificity under a sensitivity constraint of at least 90%, and the threshold yielding the highest sensitivity under a specificity constraint of at least 90%. Area under the ROC curve (AUC), sensitivity, specificity, and the selected threshold were reported for each analysis. Bootstrap confidence intervals were estimated. Internal validity of the operating characteristics was additionally assessed using optimism-corrected bootstrap (Harrell’s enhanced bootstrap, 2000 resamples). For each analysis, the number of positive and negative cases, the confusion matrix, sensitivity, specificity, positive and negative predictive values, and the Youden index are reported, each with 95% bootstrap confidence intervals (Appendix S2). Predictive values are reported as prevalence-dependent quantities given the spectrum-enriched sample.

#### Relation between IoU and usefulness

The association between IoU and usefulness was also documented (Appendix S2).

### Sensitivity analysis

Sensitivity analyses used DSC, frame-wise averaged metrics, anatomical-region subgroups, arithmetic mean aggregation of segmentation values, and the exclusion of the rater involved in the scale development.

### Statistical analysis

Analyses were performed using R 4.5.4 [R Core Team, 2026 (17)]. Continuous results were summarized using means and SDs or medians and IQRs, as appropriate. Agreement statistics were reported with their standard formulations (provided in Appendix S1), and ROC-derived thresholds were presented together with bootstrap confidence intervals. Statistical assumption checks were tested (Appendix S1).

## Results

### Study sample and data structure

**Figure 2:**
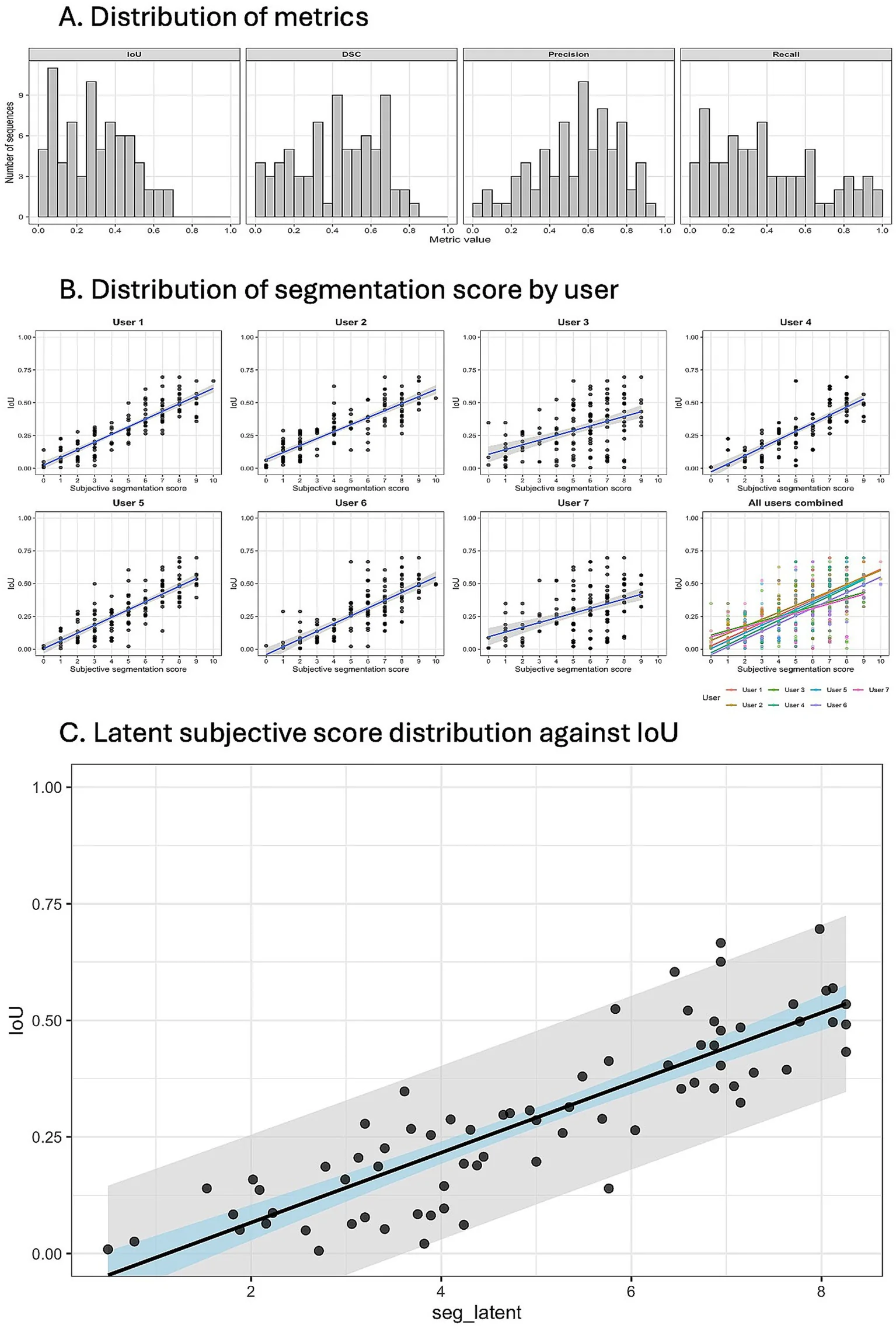
IoU and segmentation scores and seg_latent distributions. Top **(A)**: Metrics (IoU, DSC, precision, recall) distribution. Middle **(B)**: Subjective segmentation scores distribution (2 rounds) with regression lines. Bottom **(C)**: Latent segmentation score against IoU, with a regression line, 95% confidence interval (central blue ribbon), and 95% predictive interval (large grey ribbon). The confidence interval quantifies uncertainty about the average IoU expected for a given seg_latent value, whereas the prediction interval reflects the likely range of IoU values for an individual new case. IoU, Intersection over Union; DSC, Dice Similarity Coefficient.

A total of 74 ultrasound video sequences were assessed (30 popliteal sciatic; 24 brachial and 20 femoral) by seven expert anesthesiologists on two occasions, yielding 1,036 subjective segmentation ratings (16,800 single frames annotated). There were no missing data. After mixed-effects aggregation, the sequence-level analytical dataset comprised 74 observations, each with one objective IoU value, one latent subjective segmentation score (*seg_latent*), and one aggregated usefulness and usefulness_novice score (*usefulness_prop* and *usefulness_novice_prop*). Objective segmentation metrics and C-SES ratings cover a broad range (Figure 2). Visually, subjective segmentation scores were correlated with IoU with some variability. The primary results and their interpretation are given in Tables 2, 3. Detailed results are provided in Appendix S2.

**Table 3:** Step 2—Main results (determination of thresholds based on usefulness and usefulness for novices).

Domain	Variable	Analysis	Estimate	Interpretation
Reliability	Usefulness	Intra-rater Cohen’s κ, mean (SD)	0.723 SD 0.245	Substantial intra-rater agreement (but important variability especially for usefulness) Moderate inter-rater agreement
		Intra-rater Cohen’s κ, median [IQR]	0.789 IQR 0.582–0.869	
		Mean inter-rater κ	0.437 SD 0.031	
	Usefulness_novice	Intra-rater Cohen’s κ, mean (SD)	0.794 SD 0.108	
		Intra-rater Cohen’s κ, median [IQR]	0.756 IQR 0.727–0.822	
		Mean inter-rater κ	0.506 SD 0.004	
Reliability - % positive/ negative agreement	Usefulness	Intra-rater positive/negative agreement, mean (SD)	93.6% (5.2%) /78.2% (20.2%)	Positive ratings highly reproducible; negative ratings less consistently reproduced and greater between-rater variability
		Inter-rater positive/negative agreement, T1–T2	Pos: 88.1–85.3% Neg: 57.5–55.8%	Inter-rater agreement substantially higher for positive than for negative ratings, suggesting that “useful” classifications were more consistently shared across raters than “not useful” classifications
	Usefulness_novice	Intra-rater positive /negative agreement, mean (SD)	90.2% (5.6%) /89.2% (5.5%)	Positive and negative ratings similarly reproducible across repeated assessments
		Inter-rater positive/negative agreement, T1–T2	Pos: 78.0–76.6% Neg: 72.8–73.5%	Inter-rater agreement was more balanced between positive and negative classifications than for *usefulness*
Hypotheses testing for construct validity	IoU—usefulness_prop	Pearson’s r	0.735*** 95% CI [0.609; 0.825]	Strong to very strong correlation
	IoU—usefulness_novice_prop	Pearson’s r	0.830*** 95% CI [0.743; 0.890]	
Threshold determination	Usefulness proportion ≥ 0.6	ROC AUC for IoU	0.934 95% CI [0.881–0.987]	IoU Threshold = 0.206 for non-expert and 0.299 for novices Good sensitivity and specificity (> 90% for most values)
		Youden threshold for IoU	0.206 95% bootstrap CI [0.140–0.230] Se 0.807; Sp 1.000	
		ROC AUC for seg_latent	0.979 95% CI [0.949–1.000]	
		Youden threshold for seg_latent	3.510 95% bootstrap CI [3.129–4.273] Se 0.930; Sp 0.941	
	Novice usefulness proportion ≥ 0.6	ROC AUC for IoU	0.977 95% CI [0.948–1.000]	
		Youden threshold for IoU	0.299 95% bootstrap CI [0.242–0.306] Se 0.872; Sp 1.000	
		ROC AUC for seg_latent	0.987 95% CI [0.965–1.000]	
		Youden threshold for seg_latent	4.689 95% bootstrap CI [4.515–5.176] Se 0.974; Sp 0.971	

CI, Confidence interval; SD, Standard deviation; IQR, Interquartile range; IoU, Intersection over Union; Se, Sensitivity; Sp, Specificity. ***: *P* value < 0.001. Positive/negative sequence counts at the 0.6 cut-off: Non-expert usefulness 57/17, novice usefulness 39/35. Optimism-corrected estimates, confusion matrices and predictive values with 95% CIs are reported in Appendix S2.

### Step 1—Main results—validation of the C-SES

#### Mixed-effects aggregation of expert segmentation ratings

The mixed-effects model used to derive the latent segmentation score showed that between-sequence variability was the largest structured source of variance, while the rater effect was smaller and the test–retest component nested within the evaluator was limited (Table 2).

#### Concurrent criterion validity

Correlation between latent segmentation score (*seg_latent*) and IoU was very strong (*r* = 0.86; *p* < 0.001) (Table 2). In linear regression analysis, *seg_latent* was a strong predictor of IoU, and the model explained 74.1% of the variance in IoU. These results support strong concurrent criterion validity. Figure 2C shows the distribution of seg_latent with its regression line, and its 95% confidence and predictive intervals.

#### Convergent validity

Additionally, *r* between seg_latent and precision/recall was 0.498 and 0.842 (*p* < 0.001).

#### Reliability

Intra-rater (test–retest) reliability was good (ICC = 0.905). Inter-rater reliability was moderate for a single rater [ICC (2,1) = 0.689], but good to excellent when ratings were aggregated [ICC (2,3) = 0.869; ICC (2,k) = 0.94] (Table 2).

#### Measurement error

The results showed that, in the pooled single-rater analysis, the repeated single-rater C-SES scores may vary by approximately two points on the 0–10 scale (SEM 0.76, SDC 2.1, LoA − 2.4 to 1.9; all units are C-SES units). While it decreased to approximately one point when three raters were associated (SEM 0.43, SDC 1.2, LoA − 1.4 to 0.9) (Table 2).

### Step 2—secondary results—determination of thresholds

#### Reproducibility of the binary usefulness items and construct validity

For the binary *usefulness* item, intra-rater agreement was substantial (mean *κ* = 0.723 and 0.794 for novices). However, individual kappas ranged from 0.372 to 1.000 for non-expert evaluation (0.703 to 1.000 for novice evaluation), indicating significant heterogeneity between raters. Inter-rater agreement was moderate (mean κ = 0.437 and 0.506 for novices). Overall, the binary usefulness items showed acceptable reproducibility, although agreement was lower than for step 1. Hypothesis testing for construct validity showed strong to very strong association between *usefulness/usefulness_novice* and IoU (*r* = 0.735 and 0.830 for novices; *p* < 0.001). (Table 3).

#### Usefulness-based thresholds for IoU and latent segmentation

**Figure 3:**
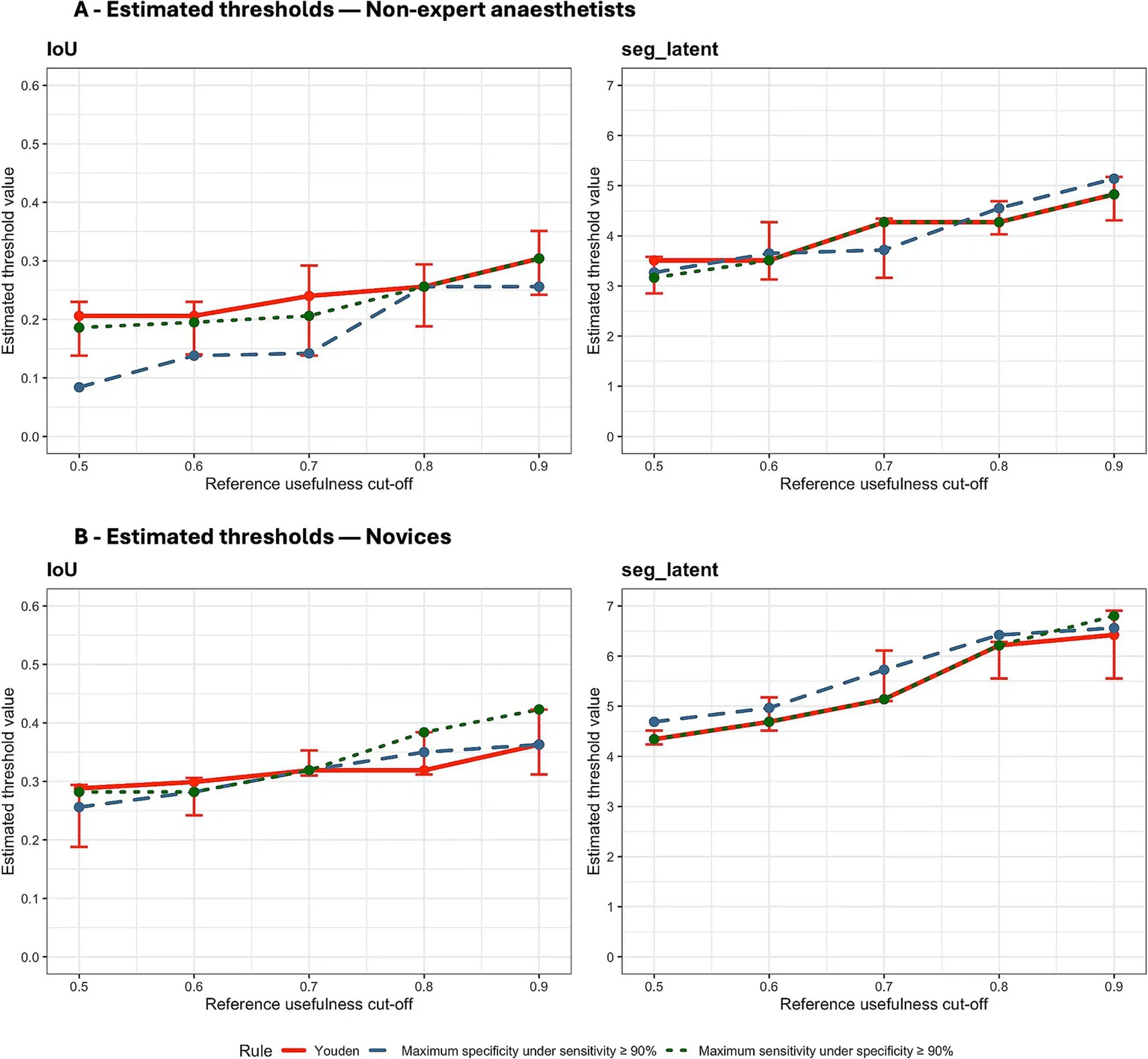
Thresholds. Top **(A)**: Thresholds for non-expert anesthesiologists. Bottom **(B)**: Thresholds for novices. All methods (Youden, specificity first, sensitivity first) and all thresholds (0.5, 0.6, 0.7, 0.8, 0.9) are represented. CI 95% is given only for Youden for clarity. IoU: Intersection over Union; seg_latent: Subjective segmentation score (aggregated).

For *usefulness*, thresholds were 0.206 for IoU and 3.51 for *seg_latent*. For *usefulness_novice*, thresholds were 0.299 for IoU and 4.689 for *seg_latent*. Almost all specificity and sensitivity were > 90% (Table 3). Figure 3 shows thresholds for alternative usefulness cut-offs (0.5, 0.7, 0.8, and 0.9) and operating rules. At the 0.6 consensus cut-off, 57 of 74 sequences were positive for non-expert usefulness (17 negative) and 39 for novice usefulness (35 negative). The sensitivity and specificity in Table 3 are apparent (uncorrected) values; internal validation by optimism-corrected bootstrap produced only minor reductions (AUC unchanged, ≤0.001; corrected Youden sensitivity/specificity 0.795/0.961 for IoU–non-expert and 0.849/0.977 for IoU–novice). Full confusion matrices, predictive values and 95% confidence intervals are reported in Appendix S2.

#### Relation between IoU and usefulness

Correlation between IoU and usefulness was strong to very strong. *R*^2^ of IoU influence on usefulness in a linear regression was 0.54 for non-experts and 0.69 for novices (Appendix S2).

### Sensitivity analysis

Sensitivity analyses were consistent with the primary findings: correlation with DSC was 0.868; region-specific correlations with IoU ranged from 0.822 to 0.895; frame-wise averaged IoU gave *r* = 0.856; and exclusion of the rater involved in scale development gave *r* = 0.838. (Appendix S2).

## Discussion

This methodological pilot study provides preliminary validation of a subjective segmentation scale for AI-based anatomy highlighting in ultrasound. The C-SES was derived from the literature and was considered clinically meaningful by expert consensus, preliminarily supporting its content validity. However, because all ratings were performed by expert regional anesthesiologists, the present validation evidence applies to expert assessors only. According to the COSMIN guidelines, the sample size of 74 sequences (> 50) was adequate (18).

The C-SES showed strong concurrent criterion validity and good reliability. However, 26% of the variance remained unexplained in the linear regression model. Moreover, inter-rater reliability was moderate for a single rater but good for three raters. In addition, the measurement error analysis showed that C-SES scores may vary by approximately two points on the 0–10 scale for a single rater, and one point for three users. These findings suggest that 1) the C-SES, when performed by an expert, is an estimation of objective segmentation quality, but does not replace IoU when precise quantitative assessment is required; it may appear more appropriate for semi-quantitative discrimination between poor, intermediate, and good segmentations than for finer distinctions at the individual-rater level and 2) grouping three experts’ ratings, as currently recommended for this purpose (19), significantly increases its robustness.

The IoU thresholds of 0.21 and 0.30 (for novice-oriented evaluation) unsurprisingly suggest that a greater degree of segmentation quality is required for novice guidance. Notably, those thresholds were substantially lower than the value of 0.5 frequently used—without any validation—in the literature as a reference point. Accordingly, the present thresholds should be regarded as context-specific and preliminary, and are not proposed as universal clinical benchmarks.

Very few studies have proposed clinically oriented segmentation thresholds in this context. Delvaux *et al.* reported an IoU of 0.49 and a DSC of 0.65 in top-rated expert-assessed videos (8), whereas Suissa *et al.* proposed a DSC threshold of 0.34 for procedural usefulness (20). Notably, this latter value was identical to that found in the present study, despite different methodologies.

This pilot study has several limitations. First, content validity was not assessed through a formal focus group or structured consensus process, and no independent expert panel was involved in the development of the scale (Appendix S3). Second, the validation sample was deliberately spectrum-enriched to span the full range of segmentation quality. Because correlation estimates depend on sample variability, the strong C-SES–IoU association and the high AUC values should be interpreted as preliminary evidence of validity within this intentionally sampled spectrum, which is expected to overestimate correlation and discrimination relative to a consecutive, unselected clinical population. Third, the study was restricted to three anatomical regions and to a single segmentation software (cNerve). External validity therefore remains uncertain, particularly for more complex regions containing multiple nerves (e.g., axillary) or for diffusion blocks (e.g., TAP or erector spinae). Fourth, the reference annotation was generated by real-time consensus annotation of a three-expert panel without prior independent annotation, exposing the process to potential groupthink bias (5). Furthermore, these three annotators were also among the seven C-SES raters, introducing partial non-independence between the reference standard and the scale under evaluation. A sensitivity analysis excluding all three annotators (leaving four fully independent raters) yielded essentially unchanged results (concurrent validity Pearson’s *r* = 0.885; inter-rater ICC and Step 2 thresholds preserved), indicating that this partial overlap did not materially affect the findings (Appendix S2). Fifth, the review of broader segmentation distributions suggested that some raters may have incorporated perceived clinical usefulness into the segmentation score, even when overlap with the reference contour was minimal. This may reinforce the need to refine the scoring instructions (including by adding a complete range of visual examples) and to clearly distinguish C-SES scoring from usefulness judgments in future validation work. In addition, “*highlighting”* performances may be replaced by “*overlap*” to make instructions more explicit. Sixth, in step 2, the choice of a 0.6 consensus cut-off and of Youden as the primary threshold rule was arbitrary. A direct clinician consensus approach (adjudication) and a threshold prioritizing sensitivity or specificity could have been chosen instead. Additional limitations of the threshold derivation step (step 2) are detailed in Appendix S3. Taken together, these considerations suggest that the derived thresholds should be regarded as hypothesis-generating.

This study has several potential implications. First, because the C-SES assesses the perceived overlap between a predicted segmentation and a target structure, its underlying construct may be relevant beyond nerve segmentation and ultrasound imaging (e.g., segmentation of other structures on CT scan or MRI). However, its degree of precision may make it a semi-quantitative tool (whose accuracy improves with multiple raters).

Second, regarding the threshold derivation step, the literature acknowledges that the relationship between metrics and their usefulness—although obviously related—is unknown (6, 21–23). Setting up thresholds based on the clinical question [and on the relative consequences of false-positive and false-negative decisions (3)] may contribute to bridging the gap between metrics and their usefulness by quantifying how much segmentation is needed to be useful for a specific clinical endpoint. However, in our study, the IoU explained only 54% of the variance of usefulness (69% for novices). The relationship between the quantitative aspect of the metrics and its usefulness should be complemented by a qualitative perspective, by expanding on the usefulness dimensions (including safety, educational value, and user confidence).

Further validation studies are necessary on a larger scale with better instructional framing, and special attention paid to variability and extreme values, following this pilot construction step. Further testing is also needed to evaluate its performance with other anatomical regions and structures, devices and annotation procedures (e.g., bounding boxes), and its transferability to other imaging modalities.

The usefulness anchors reflect expert-perceived usefulness (expert judgments about whether AI highlighting would help hypothetical non-expert or novice users). Actual non-experts and novices were not tested, and no procedural, educational, or safety outcomes were measured; therefore, the derived thresholds are not clinically validated and remain hypothesis-generating; however, they are still informative as they provide an initial order of magnitude for useful operating thresholds, and may aid in bridging the gap between metrics and usefulness.

## Data Availability

The datasets presented in this article are not readily available because data are anonymised but required authorisation from the ethic comitee to be shared. Requests to access the datasets should be directed to b.delvaux@quincyanesthesie.com.
