# Supplementary material for: Clinical scale of artificial intelligence-based nerve segmentation on ultrasound: a pilot validation study
Source: Front Med (Lausanne). 2026 Aug 20;13:1879747. doi: 10.3389/fmed.2026.1879747 (PMC13538421; doi:10.3389/fmed.2026.1879747)
Supplement: Supplementary file 1 [file Supplementary_file_1.docx]

Appendix 1 : Methods

[1. Study design	1](#_Toc235109339)

[2. Ultrasound sequences and AI model	2](#_Toc235109340)

[3. Reference standard	3](#_Toc235109341)

[4. Subjective scale	4](#_Toc235109342)

[4.1 Original scale	4](#_Toc235109343)

[4.2 Adapted scale (C-SES)	5](#_Toc235109344)

[4.3 Treatment of ordinal as continuous	6](#_Toc235109345)

[5. Rating procedure	6](#_Toc235109346)

[6. Sample size	7](#_Toc235109347)

[7. Statistical analysis	7](#_Toc235109348)

[7.1 A priori hypotheses	7](#_Toc235109349)

[7.2 Step 1 — Concurrent criterion validity	8](#_Toc235109350)

[7.3 Step 1 — Reliability	9](#_Toc235109351)

[7.4 Step 1 — Measurement error	10](#_Toc235109352)

[7.5 Step 2 — Aggregation of usefulness	10](#_Toc235109353)

[7.6 Step 2 — Dichotomisation cut-off	10](#_Toc235109354)

[7.7 Step 2 — Reliability and construct validity of binary items	11](#_Toc235109355)

[7.8 Step 2 — ROC-based threshold derivation	12](#_Toc235109356)

[8. Sensitivity analyses	14](#_Toc235109357)

[9. Software and assumption checks	14](#_Toc235109358)

[10. Ethics, data and code availability	15](#_Toc235109359)

[11. Interpretation thresholds	15](#_Toc235109360)

[References	17](#_Toc235109361)

# 1. Study design

This pilot validation study followed the COSMIN methodology and was structured in two analytical steps. Step 1 evaluated the concurrent criterion validity, reliability and measurement error of a subjective expert-rated Clinical Segmentation Evaluation Scale (C-SES) against an objective overlap metric (Intersection over Union, IoU) computed against a consensus reference standard. Step 2 explored the derivation of clinically anchored IoU thresholds, using binary expert ratings of clinical usefulness as anchors.

# 2. Ultrasound sequences and AI model

Retrospective ultrasound video sequences were used from three anatomical regions: (i) the brachial plexus at the interscalene level (C5–C6 roots and superior trunk), (ii) the femoral nerve at the inguinal crease (immediately distal to the inguinal ligament), and (iii) the sciatic nerve at the popliteal fossa (proximal to its division into tibial and common fibular components). All sequences were acquired with the Venue ultrasound system (GE HealthCare) equipped with the cNerve nerve-highlighting feature.

Predictions were generated by cNerve (GE HealthCare, Chicago USA]), a deep-learning-based nerve highlighting feature embedded in the Venue ultrasound system (R4 version). The model produces coloured overlays to highlight nerve structures in real time. Architecture of the model is not reported by the manufacturer. The model was evaluated as commercially distributed. All sequences were acquired with the dedicated regional anesthesia preset and a linear ML6-15 probe.

Sequences were drawn from a retrospective pool of regional anesthesia procedures acquired in 2025 in accordance with GRAITE-USRA guidelines^1^ in 2025 and stored for pedagogical purposes, in which the target nerve was visible throughout the recording. One of the seven evaluators pre-graded each candidate sequence into one of three segmentation-quality strata (poor, intermediate, good) for each anatomical region, prior to any rating round and blinded to all subsequent analyses. Sequences were sequentially included to fill each region-by-quality stratum with approximately equal numbers; once a stratum quota was reached, further sequences from that stratum were excluded. The pre-grading was inherently subjective and introduces a subjective component into sample composition; however, the final sample spanned a wide range of objective IoU values (Appendix 2, Figure 1), confirming successful spectrum coverage. The flow diagram of selection is shown in Figure 1. The pre-grading was purely holistic and visual (overall visual impression of segmentation quality), with no predefined numeric criteria, and these strata were used solely to enrich the sampling spectrum — not as variables in any analysis, which relied on the continuous IoU and C-SES scores. The number of sequences per region and stratum is shown in Table 1.

**Figure 1: Flow diagram of ultrasound sequence selection.** From a retrospective pool of sequences in which the target nerve was visible throughout, 112 candidate sequences were pre-graded by a single expert into three segmentation-quality strata (poor / intermediate / good) for each anatomical region. Sequences were sequentially included to fill each region-by-quality stratum with approximately equal numbers; once a stratum quota was reached, further sequences from that stratum were excluded. The final spectrum-enriched validation sample comprised 74 sequences (24 brachial plexus, 20 femoral, 30 popliteal sciatic)

| **Region** | **Poor** | **Intermediate** | **Good** | **Total** |
| --- | --- | --- | --- | --- |
| **Brachial plexus** | 6 | 9 | 9 | 24 |
| **Femoral** | 5 | 7 | 8 | 20 |
| **Popliteal sciatic** | 9 | 14 | 7 | 30 |
| **Total** | 20 | 30 | 24 | 74 |

*Table 1. Sequence counts by anatomical region and pre-grading stratum (poor / intermediate / good), n = 74.*

# 3. Reference standard

The ground truth was generated by real-time consensus annotation of a three-expert panel, without prior independent annotation. Annotation took place three weeks after the second rating round (T2). Segmentation rules followed previous methodological proposal,^2^ with the outer border of the nerve defined as the external epineurium. Annotation covered the entirety of each sequence; the ground-truth mask had to be visible throughout. Because individual pre-consensus annotations were not retained, inter-annotator agreement of the reference standard could not be quantified, and the process is potentially exposed to groupthink bias^3^.

The three annotators were also among the seven C-SES raters, introducing partial non-independence between the reference standard and the scale under evaluation. The robustness of the main findings to this overlap was explored in a sensitivity analysis excluding the rater most closely involved in scale development (Section 10).

**Computation of overlap metrics.** One frame every 50 milliseconds was extracted from each sequence and compared with the ground-truth mask to compute true positive (TP), false positive (FP) and false negative (FN) pixel counts. Metrics were then aggregated at the sequence level as follows:

$$IoU = \frac{TP}{TP + FP + FN}$$

$$DSC = \frac{2TP}{2TP + FP + FN}$$

$$Precision = \frac{TP}{TP + FP}$$

$$Recall = \frac{TP}{TP + FN}$$

As sensitivity analysis, IoU was also calculated at the image-level end then averaged for a sequence.

# 4. Subjective scale

### 4.1 Original scale

This scale was proposed by Bowness et al.[^3^](https://www.zotero.org/google-docs/?YgEeEI) :

1) Does the video contain clinically relevant images for this block area? [Y/N]

2) Rate the overall highlighting performance on a scale of 0–10? [0–very poor, 10–excellent]

3) Did the highlighting help identify the [insert structure name]? [Y/N]

4) Would the highlighting help confirm the correct ultrasound view to a less experienced practitioner? [Y/N]

### 4.2 Adapted scale (C-SES)

We used 3 of the 4 items of this scale, slightly adapted and in French. The questions were written, with examples. Further explanations were given for each request.

| **Question** | **Rating** |
| --- | --- |
| 1) Rate the overall highlighting performance on a scale of 0–10?  On a Likert scale (from 0 to 10), we evaluate the quality of cNerve’s coverage of the nerve region. Objective metrics (IoU, DSC) consider correct detections (true positives), incorrect detections (false positives), and undetected nerves (false negatives). Here, the objective is the same: to evaluate the percentage of correct work performed by the AI. We do not evaluate whether it is useful or not.  Example 1: cNerve completely covers the femoral nerve, but overlaps (too wide) -> the very high score (10/10) due to complete coverage is tempered by the fact that it extends beyond the nerve -> final score of 8/10  Example 2: cNerve covers approximately 60% of the brachial plexus and does not extend beyond it -> final score of 6/10  Example 3: cNerve covers 70% of the sciatic nerve (30% false negative), and additionally shows, at a certain point, an area that is not part of the nerve (false positive) -> final score of 5 or 6/10 | [0 - very poor, 10 - excellent] |
| **2) Does the highlighting help orient the procedure for a non-expert anesthesiologist?**  We are evaluating whether cNerve can guide the procedure performed by a certified anesthesiologist who is familiar with the basics of regional anesthesia (including the necessary anatomy), but who is not an expert. A non-expert refers to a graduated anesthesiologist who does not perform regional anesthesia on a daily basis, but has the basic knowledge of regional anesthesia (including anatomy, sonoanatomy), and has performed the 16-20 blocks per anatomical region required to meet the minimum proficiency standards.^4^  For the brachial plexus, the evaluation will focus primarily on the ability to guide the procedure for the interscalene block.  For the sciatic nerve, the focus will be primarily on the nerve prior to its division. | [Yes / No] |
| **3) Does the highlighting can help orient the procedure for a novice?**  We are evaluating whether cNerve can guide the procedures performed by a novice anesthesiologist (who lacks the fundamentals of regional anesthesia and has not performed the 16-20 blocks per anatomical region required to meet the minimum proficiency standards).[^4^](https://www.zotero.org/google-docs/?GVQLp4) | [Yes / No] |
| **Note for questions 2 and 3 :** The question is therefore: can what is shown be helpful, and is it acceptable as a means of assistance, given a certain level of knowledge? More ‘yes’ answers are expected for question 2 than for question 3 (as a novice needs clearer guidance). It is important not to turn the question on its head (‘it can’t help me because I can see the structures without the help of cNerve’) |  |

### 4.3 Treatment of ordinal as continuous

The C-SES rating was modelled as a continuous outcome in the primary analyses. This pragmatic decision is supported by Norman,^5^ who demonstrated that parametric and non-parametric methods yield near-identical inferences on Likert-type data with ≥5 categories.

# 5. Rating procedure

Seven anesthesiologists with at least five years of regional anesthesia experience served as raters. No consensual definition of “expert” exists in this field so far. Each rater scored every sequence at two rounds (T1 and T2) separated by three weeks. At each round, sequences were presented in a different randomized order, generated independently for each rater. Raters were blinded to all objective segmentation metrics, to other raters’ scores, and at T2 to their own T1 ratings. Ratings were performed in a quiet room on the same computer for all raters. Each sequence could be watched repeatedly, paused, or played backwards. A version of the sequence without the AI overlay was also available on request, to allow raters to visualize the underlying anatomy.

# 6. Sample size

Although no formal a priori power calculation was performed, the chosen sample size exceeds the COSMIN minimum of 50 and provides, post-hoc, approximately ±0.07 precision for r at r = 0.85 and ±0.10 for ICC at 0.85. With each sequence rated 14 times (7 raters × 2 rounds), the dataset includes 1,036 individual subjective ratings.

# 7. Statistical analysis

**Content validity** was first assessed qualitatively by expert consensus over the COSMIN domains of relevance, comprehensiveness, and comprehensibility (Appendix 3). The pre-specified hypotheses and analytical procedures for the remaining measurement properties are detailed below.

The content-validity appraisal was conducted as follows. During a dedicated meeting of the anaesthesia expert panel, the lead investigator first defined the key concepts underlying the instrument — segmentation, the reference (ground-truth) annotation, the AI-predicted annotation, and the notion of overlap between them. The objective overlap metrics were then explained, including the Intersection over Union (IoU) formula and its two error components (false-positive and false-negative highlighting), with illustrative examples spanning poor to good overlap. Against this shared conceptual background, the panel appraised the single C-SES item across the three COSMIN domains — relevance (the item targets the perceived overlap between AI highlighting and the nerve region of interest), comprehensiveness (a single item captures overall overlap performance rather than an isolated subcomponent), and comprehensibility (unambiguous wording in simple terms) — until consensus was reached. No structured Delphi procedure, independent panel, focus group or cognitive interviewing was performed; the resulting content-validity evidence is therefore preliminary and will require formal confirmation in future work (Appendix 3).

### 7.1 A priori hypotheses

Following the COSMIN hypothesis-testing framework, the following thresholds were pre-specified, before inspection of the data, as indicating acceptable measurement properties for the C-SES:

**Step 1 – Single-item C-SES:**

1) **Concurrent criterion validity.** Pearson’s r ≥ 0.70 (strong correlation, per Schober et al.) between *seg_latent* and IoU.
2) **Convergent construct validity.** Pearson’s r ≥ 0.40 (at least moderate, per Schober et al.) between *seg_latent* and each of the two IoU components (precision and recall).
3) **Intra- and inter-rater reliability.** ICC ≥ 0.75 (at least good reliability, per Koo & Li).
4) **Measurement error.** SEM, individual SDC, and Bland–Altman limits of agreement reported descriptively in C-SES units; no a priori numerical pass/fail threshold was pre-specified.

**Step 2 – Binary usefulness items (usefulness and usefulness_novice):**

5) **Intra- and inter-rater reliability.** Kappa ≥ 0.61 (at least substantial agreement, per Landis & Koch).
6) **Construct validity (hypothesis testing).** Pearson’s r ≥ 0.40 (at least moderate, per Schober et al.) between the aggregated usefulness proportions (*usefulness_prop* and *usefulness_novice_prop*) and IoU; r expected to be stronger for *usefulness_novice_prop* than for *usefulness_prop*.

### 7.2 Step 1 — Concurrent criterion validity

A latent segmentation value (*seg_latent*) was calculated to obtain a single value that can be correlated against IoU when assessing concurrent validity. The 0–10 subjective segmentation ratings were aggregated using a linear mixed-effects model.

If i is the patient level (i max = 74), j is the rater level (j max = 7) and k is the time level (test-retest) (k max = 2),

*s_ijk_= β_0_ + u_i_ + v_j_ + w_jk_ + ε_ijk_*

where:

- s_ijk_ the segmentation score assigned to sequence i by rater j at round k;
- β_0_ is the fixed intercept;
- u_i_ ∼ N(0,σ_u_^2^) is the random effect for sequence;
- v_j_ ∼ N(0,σ_v_^2^) is the random effect for rater;
- w_jk_ ∼ N(0,σ_w_^2^) is the random effect for rating round nested within rater;
- ε_ijk_ ∼ N(0,σ^2^) is the residual error.

The latent sequence-level subjective segmentation score was then defined as:

*seg_latent_i_= β_0_ + u_i_*

This model separated the variability attributable to the sequence itself from that related to differences between raters and test–retest variation. As a sensitivity analysis, the latent segmentation score (*seg_latent*) was replaced by the arithmetic mean of all 14 raw ratings per sequence.

The model was fitted by restricted maximum likelihood (REML) using the lme4 package (lmer) in R (bobyqa optimizer). In the derivation seg_latent(i) = β₀ + u(i), u(i) is the empirical Bayes (best linear unbiased predictor, BLUP) conditional mode of the sequence random effect; seg_latent(i) therefore represents the model-estimated expected C-SES score for sequence i at an average rater and rating round, on the original 0–10 scale.

### 7.3 Step 1 — Reliability

**Intra-rater reliability** of the C-SES was assessed by computing one single-rater test–retest ICC per evaluator, applied to each evaluator's pair of ratings on the same set of sequences (T1 vs T2). To summarise across the seven evaluators, we report the mean, standard deviation, median, and interquartile range of the seven individual estimates.

We chose this descriptive aggregation rather than fitting a single global mixed model jointly across raters, sequences, and time, for two reasons. First, reporting the mean and dispersion of the seven separate estimates conveys both the central reliability and its heterogeneity across evaluators — an aspect that a pooled estimate would hide. Second, this approach is conservative: it reflects the reliability that any individual clinician using the C-SES would experience on average, rather than an idealised scale-level reliability that no single user would ever observe in practice. We did not compute a confidence interval for the mean across raters, as combining seven non-independent estimates into a mean-of-means interval would understate the true uncertainty without a formal hierarchical bootstrap; the dispersion statistics convey heterogeneity instead.

A two-way random-effects absolute-agreement ICC was pre-specified, reported as ICC(2,1) (single rater), ICC(2,3) (mean of three raters) and ICC(2,k=7) (mean of all seven raters). **Inter-rater reliability.** ICCs were computed in three complementary ways: at T1 alone, at T2 alone, and on the within-rater mean of T1 and T2. The third estimate is the *primary* inter-rater estimate, as averaging across rounds reduces within-rater noise before estimating the between-rater component. Importantly, this primary estimate is the ICC computed on within-rater mean ratings, not the arithmetic mean of the two single-round ICCs; the single-round estimates are reported alongside it for transparency.

### 7.4 Step 1 — Measurement error

Measurement error was assessed as a complement to test–retest reliability. Whereas ICCs quantify relative reliability, measurement error indices describe the absolute variability of repeated scores in the original unit of the scale. For each rater, paired C-SES scores from the first and second rating rounds were used to calculate the test–retest difference. The standard error of measurement (SEM) was calculated as the standard deviation of the differences divided by $\sqrt{2}$. The individual smallest detectable change (SDC) was calculated as $1.96 x \sqrt{2} x SEM$. Bland–Altman limits of agreement (LoA) were calculated as the mean difference between rounds ± 1.96 times the standard deviation of the differences. These indices were calculated separately for each rater and in a pooled analysis including all complete patient–rater pairs.

Because the intended use of the scale may involve aggregated expert ratings, measurement error was also estimated for the mean of three raters. All possible combinations of three raters among the seven experts were generated. For each combination, C-SES scores were averaged separately at the first and second rating rounds for each sequence. Test–retest differences between the two three-rater mean scores were then used to calculate the SEM, individual SDC, and Bland–Altman limits of agreement. Results were summarized across the 35 possible three-rater combinations and interpreted descriptively

### 7.5 Step 2 — Aggregation of usefulness

Each sequence’s binary usefulness ratings were aggregated as the observed proportion of positive ratings (*usefulness_prop* and *usefulness_novice_prop*). This proportion-based approach was preferred over a model-based latent probability because it is directly interpretable and free of modelling assumptions. Both estimators were computed and found to be effectively identical (Pearson r = 1.00); the latent-probability version was retained as a sensitivity analysis.

### 7.6 Step 2 — Dichotomisation cut-off

A sequence was classified as clinically useful when the proportion of positive ratings reached or exceeded 0.6 — a clear majority that is more demanding than a simple 0.5 threshold. This cut-off is acknowledged as arbitrary and was pre-specified before any analysis. Sensitivity analyses repeated the entire threshold derivation with cut-offs of 0.5, 0.7, 0.8 and 0.9, applied symmetrically to both binary anchors.

### 7.7 Step 2 — Reliability and construct validity of binary items

The threshold derivation in Step 2 takes the binary usefulness ratings as inputs. Although a full COSMIN validation pipeline was beyond the scope of the present pilot study, we used available data to verify that these binary ratings carried adequate measurement properties. We therefore conducted a focused assessment of intra-rater reliability, inter-rater reproducibility, and construct validity for both the expert usefulness item (*usefulness*) and the novice-oriented item (*usefulness_novice*). The same methodology was applied to both items in parallel, and results are reported separately for each.

#### Intra-rater reliability

Each expert rated every sequence twice (T1 and T2). For each rater, an unweighted Cohen's kappa was computed. The seven kappa coefficients were then summarised by the mean (SD) and the median (IQR). The rationale for this descriptive aggregation rather than a pooled model is the same as for the C-SES intra-rater reliability described elsewhere in this appendix.

#### Inter-rater reproducibility

Inter-rater reliability was assessed separately at each rating round. Because all raters evaluated the same set of sequences, a multi-rater kappa approach was used. Specifically, exact multi-rater kappa based on Conger’s extension was calculated at the first and second rating rounds for each binary anchor. Kappa values were reported separately for each round, together with a descriptive mean across rounds. The mean across rounds was used only as a descriptive summary and was not interpreted as an independent reliability coefficient.

We report the multi-rater kappa at the first round (T1) and at the second round (T2) separately, together with a descriptive arithmetic mean of the two values. The descriptive mean is reported as a convenience summary and does not constitute a formal aggregated estimator; it should be interpreted as approximating the inter-rater agreement that would be observed in a typical single rating session, averaged over the two rounds available in the present pilot.

#### Positive and negative agreement

To complement the kappa-based statistics, we also computed the **percentage of positive agreement** and the **percentage of negative agreement**. These quantities are particularly informative when the two rating categories are unbalanced, because Cohen's kappa is known to be sensitive to marginal prevalence (the so-called "kappa paradox"), and a low kappa can coexist with very high observed agreement when one category dominates. Reporting positive and negative agreement explicitly therefore allows the reader to distinguish a genuine reproducibility problem from a low-kappa artefact driven by class imbalance — a distinction that is directly relevant here, given the high prevalence of "useful" classifications in the dataset.

The percentage of positive agreement was computed as the proportion of paired ratings in which both observers classified the sequence as "useful", among all pairs in which at least one observer did so; the percentage of negative agreement was computed analogously for the "not useful" category.

#### Hypothesis testing for construct validity

To assess whether the aggregated usefulness scores measure a construct that aligns with objective segmentation quality, we computed Pearson's correlation between the proportion-based aggregated reference (*usefulness_prop* and *usefulness_novice_prop*) and the IoU of the corresponding sequence. We pre-specified that a moderate positive correlation was expected if the construct is valid, on the rationale that sequences with higher overlap between the AI-generated mask and the ground truth should be judged more clinically useful by experts and novices alike. Furthermore, we prespecified a stronger positive correlation for *usefulness_novice_prop* than for *usefulness_prop*.

This analysis falls within the **hypothesis-testing framework for construct validity**, in the sense of the COSMIN taxonomy: a measurement instrument is supported by construct validity when its scores correlate, in pre-specified directions and magnitudes, with measures of related but non-identical constructs. Here, IoU is a related but non-identical construct - it captures objective spatial overlap but probably does not cover all the aspects of clinical usefulness - and the expected correlation is moderate to strong, consistent with the partial overlap between the two constructs. The hypothesis was specified before inspecting the results. The same correlation was computed separately for *usefulness_prop* and *usefulness_novice_prop*.

### 7.8 Step 2 — ROC-based threshold derivation

As mentioned earlier in this appendix, for the threshold analyses, the aggregated binary usefulness variables were used as clinical reference standards. Two references were considered separately: the non-expert-oriented reference, derived from *usefulness_prop*, and the novice-oriented reference, derived from *usefulness_novice_prop*. In the primary analysis, a sequence was classified as clinically useful when the corresponding usefulness proportion was at least 0.6. Alternative cut-offs of 0.5, 0.7, 0.8 and 0.9 were explored in sensitivity analyses.

For each clinical reference, ROC analyses were performed separately for the objective overlap metric and for the latent subjective segmentation score. In the main analysis, the continuous predictors were therefore IoU and *seg_latent*. Higher values of both predictors were assumed to indicate better segmentation quality and, consequently, a greater probability of being classified as clinically useful.

Sensitivity was defined as the proportion of clinically useful sequences correctly identified above a given threshold, whereas specificity was defined as the proportion of non-useful sequences correctly identified below that threshold. The area under the ROC curve was used to quantify the overall discriminative ability of each predictor against the binary clinical reference.

The primary threshold selection rule was the Youden index, which identifies the threshold providing the best combined balance between sensitivity and specificity. This rule was selected because false-positive and false-negative classifications were considered to be of comparable importance in the primary analysis. Two additional operating rules were reported to illustrate clinically oriented alternatives. First, among thresholds achieving sensitivity of at least 90%, the threshold with the highest specificity was selected. Second, among thresholds achieving specificity of at least 90%, the threshold with the highest sensitivity was selected. These secondary analyses were intended to show how the selected threshold would change if the clinical priority were to minimise false negatives or false positives.

Uncertainty was estimated using percentile bootstrap resampling with 2,000 sequence-level resamples. Resampling was stratified by the binary reference class to preserve the number of clinically useful and non-useful sequences in each bootstrap sample. For each bootstrap replicate, the ROC analysis and the full threshold selection procedure were repeated. Bootstrap confidence intervals were obtained from the empirical 2.5th and 97.5th percentiles of the bootstrap distributions. If, for a given subgroup or cut-off, the binary reference contained only one class, ROC-derived thresholds were considered not estimable and were reported as such.

The same procedure was applied to the primary non-expert and novice references and repeated in sensitivity analyses using alternative usefulness cut-offs and alternative objective metrics. Candidate thresholds were interpreted as internally derived operating points rather than definitive clinical decision rules, pending more formal validation of the usefulness concepts.

Internal validity of the derived operating points was assessed using an optimism-corrected bootstrap (Harrell’s enhanced bootstrap, 2000 resamples). In each resample the threshold-selection rule was re-estimated and applied both to the resample (apparent performance) and to the original sample (test performance); the optimism was taken as the mean apparent-minus-test difference and subtracted from the apparent estimate (corrected = apparent − optimism). Because discrimination is based on a single raw metric rather than a fitted multivariable model, the AUC is not subject to threshold-selection optimism and its correction is expected to be negligible. For each analysis we additionally report the number of positive and negative cases, the confusion matrix (true and false positives and negatives), and the positive and negative predictive values, each with 95% bootstrap confidence intervals. Predictive values are prevalence-dependent and, given the deliberately spectrum-enriched sample, are not interpreted as clinical predictive values.

# 8. Sensitivity analyses

Sensitivity analyses were performed to assess the robustness of the main findings across alternative analytical assumptions and data subsets:

- The main analyses were repeated using an alternative IoU aggregation strategy: whereas the primary analysis used IoU computed at the sequence level, the sensitivity analysis used frame-wise IoU values averaged at the sequence level.
- The main criterion-validity and threshold analyses were repeated using DSC instead of IoU as the objective overlap metric.
- Analyses were repeated separately within each anatomical region — femoral, sciatic and brachial — in order to explore whether the association between the C-SES and objective segmentation metrics was consistent across anatomical contexts.
- Analyses were repeated using alternative usefulness cut-offs of 0.5, 0.7, 0.8 and 0.9, in addition to the primary cut-off of 0.6. When a subgroup or cut-off produced a binary reference with only one class, ROC-based thresholds were considered not estimable and were reported as such.
- Because one rater had contributed to the development of the measurement approach, the main analyses were repeated after excluding this rater to explore potential development-related bias.
- Replacement of *seg_latent* by the arithmetic mean of all 14 raw ratings per sequence (Section 8.2).
- Replacement of *usefulness_prop* by the model-based latent probability (Section 8.5).

These sensitivity analyses were intended to evaluate internal robustness rather than establish external validity, which will require independent datasets and additional devices

# 9. Software and assumption checks

All analyses were performed in R version 4.5.4 (R Foundation for Statistical Computing, Vienna, Austria) in RStudio version 2026.01.0+392. Used R packages were: rmarkdown, knitr, dplyr, tidyr, lme4, performance, car, lmtest, ggplot2, patchwork, irr, pROC, report.

For Pearson’s correlation, the relationship was assessed for approximate linearity, the absence of major influential observations, and univariate normality on Shapiro–Wilk. For Spearman’s correlation, the relationship was assessed for monotonicity and the absence of major influential observations.

For linear regression, we checked linearity, independence, homoscedasticity, normality of residuals, absence of influential outliers, and lack of excessive multicollinearity.

# 10. Ethics, data and code availability

The study was approved by a national ethics comite (IRB number IRB00010835). Ultrasound sequences were anonymized before analysis, and no patient identifiers were accessible to raters or analysts.

The ultrasound sequences themselves cannot be publicly shared because of patient data-protection constraints and manufacturer agreements; reasonable requests for collaborative re-analysis can be addressed to the corresponding author.

# 11. Interpretation thresholds

**Correlations (Pearson’s r, Spearman’s ρ) were interpreted using the Schober et al. benchmarks^6^ :**

| 0.00–0.10 | Negligible correlation |
| --- | --- |
| 0.10–0.39 | Weak correlation |
| 0.40–0.69 | Moderate correlation |
| 0.70–0.89 | Strong correlation |
| 0.90–1.00 | Very strong correlation |

**Cohen’s kappa was interpreted using the Landis and Koch benchmarks^7^ :**

| 0.00–0.20 | Slight |
| --- | --- |
| 0.21–0.40 | Fair |
| 0.41–0.60 | Moderate |
| 0.61–0.80 | Substantial |
| 0.81–1.00 | Almost perfect |

**Intraclass correlation coefficients (ICCs) were interpreted using the Koo and Li benchmarks^8^ :**

| < 0.50 | Poor |
| --- | --- |
| 0.50–0.75 | Moderate |
| 0.75–0.90 | Good |
| > 0.90 | Excellent |

*Table 2 : Interpretation thresholds according to litterature*

# References

1.	Zhang X, Zhang L, Wang X, Wu Z. Evaluation of AI-based nerve segmentation on ultrasound. Comment on Br J Anaesth 2025; 134: 1497-502. *Br J Anaesth*. 2025;135(6):1847-1848. doi:10.1016/j.bja.2025.08.033

2.	Delvaux BV, Guessous K, Bowness JS, et al. Artificial intelligence-based nerve segmentation: standardised methodology needed for ultrasound-guided regional anaesthesia. *Br J Anaesth*. 2025;135(3):795-797. doi:10.1016/j.bja.2025.05.052

3.	Chen PHC, Mermel CH, Liu Y. Evaluation of artificial intelligence on a reference standard based on subjective interpretation. *The Lancet Digital Health*. 2021;3(11):e693-e695. doi:10.1016/S2589-7500(21)00216-8

4.	Chuan A, Jeyaratnam B, Fathil S, et al. Non-Fellowship regional anesthesia training and assessment: an international Delphi study on a consensus curriculum. *Reg Anesth Pain Med*. 2021;46(10):867-873. doi:10.1136/rapm-2021-102934

5.	Norman G. Likert scales, levels of measurement and the “laws” of statistics. *Adv Health Sci Educ Theory Pract*. 2010;15(5):625-632. doi:10.1007/s10459-010-9222-y

6.	Schober P, Boer C, Schwarte LA. Correlation Coefficients: Appropriate Use and Interpretation. *Anesthesia & Analgesia*. 2018;126(5):1763. doi:10.1213/ANE.0000000000002864

7.	Landis JR, Koch GG. The measurement of observer agreement for categorical data. *Biometrics*. 1977;33(1):159-174.

8.	Koo TK, Li MY. A Guideline of Selecting and Reporting Intraclass Correlation Coefficients for Reliability Research. *Journal of Chiropractic Medicine*. NaN/NaN/NaN;15(2):155-163. doi:10.1016/j.jcm.2016.02.012
