# Supplementary material for: Clinical scale of artificial intelligence-based nerve segmentation on ultrasound: a pilot validation study
Source: Front Med (Lausanne). 2026 Aug 20;13:1879747. doi: 10.3389/fmed.2026.1879747 (PMC13538421; doi:10.3389/fmed.2026.1879747)
Supplement: Supplementary file 2 [file Supplementary_file_2.docx]

Appendix 2 – Results

[Data completeness and descriptive statistics	1](#_Toc235109315)

[Univariate distributions of IoU and segmentation	2](#_Toc235109316)

[Bivariate distribution of IoU and segmentation	4](#_Toc235109317)

[Univariate distribution of usefulness	5](#_Toc235109318)

[Step 1: Validation of the C-SES	7](#_Toc235109319)

[Mixed-effects derivation of the latent C-SES score	8](#_Toc235109320)

[Concurrent criterion validity against IoU	9](#_Toc235109321)

[Hypotheses testing for construct validity	9](#_Toc235109322)

[Intra-rater reliability	10](#_Toc235109323)

[Inter-rater reliability	10](#_Toc235109324)

[Measurement error	10](#_Toc235109325)

[Step 2: usefulness anchors and threshold derivation	12](#_Toc235109326)

[Reliability of the binary usefulness anchors	13](#_Toc235109327)

[Relationship between IoU and usefulness	14](#_Toc235109328)

[Threshold results	14](#_Toc235109329)

[Sensitivity analyses	17](#_Toc235109330)

[Alternative objective metric: DSC	20](#_Toc235109331)

[Region-specific analyses	20](#_Toc235109332)

[Sequence-level versus frame-level aggregation	21](#_Toc235109333)

[Sensitivity analysis using the arithmetic mean of ratings	21](#_Toc235109334)

[Sensitivity analysis using latent usefulness probabilities	21](#_Toc235109335)

[Exclusion of overlapping raters: the scale-development rater and the reference annotators	21](#_Toc235109336)

[Overall interpretation of supplementary results	22](#_Toc235109337)

[Assumption checks	22](#_Toc235109338)

This appendix complements the main manuscript with detailed numerical results, auxiliary tables and sensitivity analyses. It is organised in four blocks: (1) data completeness and descriptive statistics; (2) Step 1 — full validation of the C-SES (mixed-effects derivation of seg_latent, concurrent and convergent validity, reliability, measurement error); (3) Step 2 — full results of the usefulness anchors and ROC-based threshold derivation; and (4) sensitivity analyses (alternative metric DSC, anatomical regions, frame-level aggregation, arithmetic-mean aggregation, latent usefulness probabilities, exclusion of the rater involved in scale development) and statistical assumption checks. All figures referenced here are reproduced in the supplementary figures.

# Data completeness and descriptive statistics

The analytical dataset included 74 ultrasound video sequences. Each sequence was rated by seven expert anaesthetists on two occasions, yielding 1,036 expected subjective segmentation ratings. The same structure was used for the binary usefulness ratings (Table 1). Objective segmentation metrics were available at sequence level for all included sequences. No imputation was therefore required for the main analyses. Demographic characteristics are described in Table 2.

Sequence inclusion proceeded as follows. From a retrospective pool of clinical ultrasound recordings, 112 candidate sequences were pre-graded by a single expert into three segmentation-quality strata (poor, intermediate, good) for each anatomical region. Sequences were sequentially included to fill each region-by-quality stratum with approximately balanced numbers; once a stratum quota was reached, further sequences from that stratum were excluded. The final spectrum-enriched validation sample comprised 74 sequences (24 brachial plexus, 20 femoral, 30 popliteal sciatic).

| **Data type** | **Expected observations** | **Available observations** | **Completeness** |
| --- | --- | --- | --- |
| Segmentation ratings | 74 × 7 × 2 = 1036 | 1036 | 100% |
| usefulness ratings | 1036 | 1036 | 100% |
| usefulness_novice ratings | 1036 | 1036 | 100% |
| Objective metrics | 74 | 74 | 100% |

#### Table 1: Data completeness

|  | **Estimate (standard deviation)** |
| --- | --- |
| **Age (years)** | 63 (18.4) |
| **Weight (kg)** | 76 (16.4) |
| **Size (cm)** | 167.5 (9.4) |
| **Body-Mass Index (BMI)** | 27.7 (5.5) |

#### Table 2 : Demographic characteristics

### Univariate distributions of IoU and segmentation

Descriptive distributions showed that objective segmentation performance varied substantially across sequences and anatomical regions (Figure 1). This variability was important for validation, as it allowed the relationship between the subjective C-SES score and the objective reference metrics to be assessed across a broad range of segmentation quality. The raw C-SES ratings also covered a wide part of the 0–10 scale (Figure 2), indicating that raters used the scale with sufficient spread. The distribution of the latent C-SES score was consistent with this pattern (Figure 3) and supported its use as a sequence-level summary of perceived segmentation quality.

#### Figure 1: Distribution of metrics (overall and by regions)

#### Figure 2: Distribution of subjective segmentation score (overall, by rounds, and by raters and rounds)

#### Figure 3 : Distribution of the latent segmentation score

### Bivariate distribution of IoU and segmentation

User-specific scatterplots showed a positive association between C-SES ratings and IoU for all raters, supporting the overall concurrent validity results (Figure 4). However, the dispersion and slope of the user-specific regression lines varied, suggesting differences in rater calibration. Some raters showed a more linear relationship with IoU, whereas others showed greater dispersion and occasional high C-SES scores despite very low IoU values.

#### Figure 4: relationship between IoU and subjective segmentation score by user

Individual users (User 1 to 7) and combined plot with user specific regression-line. T1 and T2 was pooled for each graph (74 x 2 = 148 points)

A post hoc review of highly discordant cases identified one specific sequence as the clearest example. This sequence had near-zero overlap (IoU 0.0057; DSC 0.0113) but received C-SES scores of 6 or higher from two raters at both rating rounds. Video review confirmed extensive false-positive highlighting with almost no true-positive overlap, suggesting that false-positive regions were interpreted as clinically meaningful segmentation. Such cases were retained in the analyses and interpreted as part of the expected discrepancy between subjective clinical judgement and strict overlap-based metrics.

The review of discordant cases and broad distribution also suggested that, in rare instances, raters may have incorporated perceived clinical usefulness into the segmentation score, even when overlap with the reference contour was minimal. This reinforces the need to refine the scoring instructions and to clearly distinguish C-SES scoring from usefulness judgements in future validation work.

### Univariate distribution of usefulness

The aggregated usefulness proportions also showed meaningful variability (Figure 5, Tables 3 & 4). At the primary cut-off of 0.6, 57 sequences were classified as useful for a non-expert anaesthesiologist and 39 as useful for a novice. Across the sensitivity cut-offs, the proportion of positive classifications decreased as expected, confirming that stricter usefulness definitions produced progressively more selective clinical references.

| **variable** | **n** | **mean (SD)** | **median [IQR]** | **min-max** | **n (%) >= 0.6** |
| --- | --- | --- | --- | --- | --- |
| **usefulness_prop** | 74 | 0.77 (0.30) | 0.89 [0.36] | 0.00-1.00 | 57 (77.0%) |
| **usefulness_novice_prop** | 74 | 0.54 (0.37) | 0.64 [0.77] | 0.00-1.00 | 39 (52.7%) |

#### Table 3 : Central and dispersion values of usefulness proportions for the cut-off of 0.6

#### Figure 5 : Distribution of aggregated usefulness proportions

Dashed line indicates the primary reference cut-off of 0.6

| **cut_off** | **usefulness_prop: n (%) positive** | **usefulness_novice_prop: n (%) positive** |
| --- | --- | --- |
| **0,5** | 62 (83.8%) | 43 (58.1%) |
| **0,6** | 57 (77.0%) | 39 (52.7%) |
| **0,7** | 54 (73.0%) | 32 (43.2%) |
| **0,8** | 44 (59.5%) | 25 (33.8%) |
| **0,9** | 37 (50.0%) | 20 (27.0%) |

#### Table 4 : Usefulness proportion above different cut-offs

# Step 1: Validation of the C-SES

Results will be reviewed and explained in the text and are also provided in Table 5.

| **Domain** | **Variables** | **Analysis** | **Estimate** | **95% CI** | **p value / Other information** |
| --- | --- | --- | --- | --- | --- |
| **Latent score derivation** | seg_latent | Mixed model intercept | 5.017 | - |  |
|  |  | SD(patient) | 2.104 | - |  |
|  |  | SD(evaluator) | 0.471 | - |  |
|  |  | SD  (evaluator:retest) | 0.183 | - |  |
|  |  | Residual SD | 1.374 | - |  |
| **Concurrent validity** | IoU – seg_latent | Pearson r | 0.861 | [0.787 ; 0.910] | p < .001 |
|  |  | Spearman rho | 0.866 | [0.804 ; 0.904] | p < .001 |
|  |  | Linear regression slope β | 0.075 | [0.065 ; 0.085] | p < .001  R²=0.7405; RSE=0.0926 |
| **Hypothese testing for Construct validity** | Precision – seg_latent | Pearson r | 0.498 | [0.305 ; 0.653] | p < .001 |
|  | Recall – seg_latent | Pearson r | 0.842 | [0.759 ; 0.897] | p < .001 |
| **Reliability** | C-SES | Intra-rater ICC(2,1), mean | 0.905 | - | SD 0.032 |
|  |  | Intra-rater ICC(2,1), median | 0.899 | - | IQR 0.035 |
|  |  | Intra-rater ICC(2,1), range | 0.850 - 0.940 |  |  |
|  |  | Inter-rater ICC(2,1), 1st pass | 0.695 | [0.613; 0.773] |  |
|  |  | Inter-rater ICC(2,1), 2d pass | 0.623 | [0.528; 0.714] |  |
|  |  | Inter-rater ICC(2,1), 2 passes | 0.689 | [0.602 ; 0.770] |  |
|  |  | Inter-rater ICC(2,3), 1st pass | 0.872 | [0.826; 0.911] |  |
|  |  | Inter-rater ICC(2,3), 2d pass | 0.831 | [0.770; 0.883] |  |
|  |  | Inter-rater ICC(2,3), 2 passes | 0.869 | [0.820 ; 0.909] |  |
|  |  | Inter-rater ICC(2,k=7), 1st pass | 0.941 | [0.917; 0.960] |  |
|  |  | Inter-rater ICC(2,k=7), 2d pass | 0.920 | [0.886; 0.946] |  |
|  |  | Inter-rater ICC(2,k=7), 2 passes | 0.94 | [0.914 ; 0.959] |  |
| **Measurement error** | C-SES (pooled) | Mean test-retest bias | -0.26  T1-T2 pooled analysis |  |  |
|  |  | SEM | 0.76 CES-points |  |  |
|  |  | individual SDC | 2.12  CES-points |  |  |
|  |  | Bland–Altman LoA | −2.38 to +1.86 |  |  |
|  | C-SES (per-rater summary) | SEM mean | 0.740 |  | SD 0.141 |
|  |  | SEM median | 0.724 |  | IQR 0.184 |
|  |  | SDC mean | 2.051 |  | SD 0.391 |

#### Table 5 – step 1 (main analysis)

**Interpretation note.** ICC(2,1) reflects the reliability of a single rater scoring a sequence; ICC(2,3) corresponds clinically to a three-rater consensus (e.g. three experts averaging their scores); ICC(2,k=7) corresponds to the average of all seven raters and represents the theoretical maximum reliability achievable with this panel. The progressive increase from ICC(2,1) to ICC(2,k=7) quantifies the gain in reliability from multi-rater aggregation.

### Mixed-effects derivation of the latent C-SES score

The mixed-effects aggregation model showed that the largest structured source of variability was the sequence effect. The standard deviation for the sequence random effect was 2.104, compared with 0.471 for the evaluator effect and 0.183 for the evaluator-by-retest component. The residual standard deviation was 1.374. This indicates that C-SES ratings were primarily driven by differences in perceived segmentation quality between sequences, rather than by systematic differences between raters or by the rating round.

The fixed intercept was 5.017, representing the overall average C-SES level. The latent sequence-level C-SES score was therefore derived as the fixed intercept plus the sequence-specific random effect. This modelling strategy produced a score reflecting the perceived segmentation quality of each sequence, adjusted for rater severity and test–retest effects.

Figure 6 shows the distribution of the subjective latent (aggregated) score against IoU, with regression line, 95% confidence and predictive intervals. The confidence interval quantifies uncertainty about the average IoU expected for a given *seg_latent* value, whereas the prediction interval reflects the likely range of IoU values for an individual new case.

#### Figure 6: Distribution of the subjective latent (aggregated) score against IoU

Line = regression line; central blue ribbon = 95% confidence interval; large grey ribbon = 95 % predictive interval (large grey ribbon)

### Concurrent criterion validity against IoU

The C-SES showed strong concurrent criterion validity against IoU. Pearson’s correlation was 0.861, with a 95% CI from 0.787 to 0.910, and Spearman’s rho was 0.866, with a 95% CI from 0.804 to 0.904 (p < .001). The similarity between Pearson and Spearman coefficients suggests that the association was both strong and robust to rank-based assessment.

The linear regression slope was 0.075, with a 95% CI from 0.065 to 0.085, meaning that each one-point increase in latent C-SES was associated with an average increase of approximately 0.075 in IoU. The model explained 74.1% of the variance in IoU, with a residual standard error of 0.0926. These results indicate that the C-SES is a strong proxy for objective segmentation quality. However, the remaining unexplained variance (25.9%) suggests that the C-SES should not be interpreted as a strict substitute for IoU.

### Hypotheses testing for construct validity

Associations with precision and recall were interpreted as hypotheses testing for construct validity, because these metrics are related to segmentation quality but were not treated as reference annotations. Precision was more moderately associated with C-SES, with Pearson’s r of 0.498. Recall showed a stronger association, with Pearson’s r of 0.842. This pattern suggests that expert perception of segmentation quality was more closely aligned with the extent to which the target structure was recovered than with precision alone.

### Intra-rater reliability

Intra-rater reliability was excellent. The mean ICC(2,1) across the seven raters was 0.905, with an SD of 0.032. The median was 0.899, with an IQR of 0.035, and individual ICCs ranged from 0.850 to 0.940. This narrow range indicates consistently high test–retest reproducibility across raters.

| **Rater** | **ICC value** |
| --- | --- |
| **Rater 1** | 0.916 |
| **Rater 2** | 0.891 |
| **Rater 3** | 0.896 |
| **Rater 4** | 0.940 |
| **Rater 5** | 0.850 |
| **Rater 6** | 0.899 |
| **Rater 7** | 0.940 |

#### Table 6 – Individual intra-rater ICC by raters

### Inter-rater reliability

Inter-rater reliability was moderate for a single rater but good to excellent when ratings were aggregated. For a single rater, ICC(2,1) was 0.695 at the first pass and 0.623 at the second pass. After averaging both passes for each patient–rater pair, ICC(2,1) was 0.689. Thus, single-rater inter-rater reliability was moderate, with slightly lower agreement at the second pass but broadly similar results across the two rounds.

For the projected mean of three raters, ICC(2,3) was 0.872 at the first pass, 0.831 at the second pass, and 0.869 after averaging both passes. These values indicate good reliability for a clinically realistic three-rater aggregate. For the mean of all seven raters, ICC(2,k=7) was 0.941 at the first pass, 0.920 at the second pass, and 0.940 after averaging both passes. This confirms that multi-rater aggregation substantially improves reliability compared with single-rater scoring.

### Measurement error

**Measurement-error analyses** complemented the ICC findings by quantifying absolute error in C-SES points. In the pooled single-rater test–retest analysis, the mean bias was −0.26 points, indicating slightly lower ratings at the second pass. The SEM was 0.76 points and the individual SDC was 2.12 points. Thus, repeated single-rater C-SES scores may vary by approximately two points on the 0–10 scale (Table 2). Individual error measurement are shown in Table 4.

| **Rater** | **Bias T2–T1** | **SEM** | **SDC individual** | **LoA** |
| --- | --- | --- | --- | --- |
| 1 | −0.46 | 0.72 | 2.00 | −2.46 to +1.54 |
| 2 | −0.35 | 0.93 | 2.58 | −2.94 to +2.24 |
| 3 | −0.26 | 0.77 | 2.13 | −2.39 to +1.88 |
| 4 | −0.15 | 0.57 | 1.58 | −1.73 to +1.43 |
| 5 | −0.57 | 0.89 | 2.47 | −3.04 to +1.90 |
| 6 | −0.22 | 0.72 | 2.01 | −2.22 to +1.78 |
| 7 | +0.19 | 0.57 | 1.58 | −1.39 to +1.77 |

#### Table 7: Measurement error by rater

Individual error measurement

Measurement error was lower when scores were **averaged across three raters**. Across all possible three-rater combinations, the mean SEM was 0.43, and the mean SDC was 1.18. The average lower and upper limits of agreement were approximately −1.44 and +0.92 points, respectively. This supports the conclusion that multi-rater aggregation reduces measurement error and provides a more stable estimate of perceived segmentation quality (Table 6).

| Parameter | Mean (SD) | Median [IQR] | Min–max |
| --- | --- | --- | --- |
| Bias (T2–T1) | −0.26 (0.11) | −0.27 [0.17] | −0.46 to −0.06 |
| SEM for 3-rater mean | 0.43 (0.05) | 0.42 [0.07] | 0.34 to 0.53 |
| SDC for 3-rater mean | 1.18 (0.13) | 1.17 [0.19] | 0.94 to 1.47 |
| Lower LoA | −1.44 (0.23) | −1.45 [0.37] | −1.93 to −1.04 |
| Upper LoA | 0.92 (0.08) | 0.93 [0.14] | 0.77 to 1.05 |

#### Table 8: Measurement error of the C-SES averaged across three raters

SEM: standard error of measurement; SDC: smallest detectable change; LoA: Bland–Altman limits of agreement.

Estimates were calculated empirically across all possible combinations of three raters among the seven available raters. For each three-rater combination, C-SES scores were averaged at T1 and T2, and test–retest differences were used to estimate measurement error. The 35 combinations are not statistically independent and should therefore be interpreted descriptively.

# Step 2: usefulness anchors and threshold derivation

Results will be reviewed and explained in the text and are also provided in Table 8.

| **Domain** | **Comparator/**  **outcome** | **Analysis** | **Estimate** | **95% CI /**  **dispersion** | **p value/ Other information** |
| --- | --- | --- | --- | --- | --- |
| **Reliability** | usefulness | Intra-rater Cohen’s κ, mean | 0.723 | SD 0.245 |  |
|  |  | Intra-rater Cohen’s κ, median | 0.789 | IQR [0.582–0.869] |  |
|  |  | Inter-rater κ, 1st pass | 0.458 |  |  |
|  |  | Inter-rater κ, 2d pass | 0.415 |  |  |
|  |  | Inter-rater κ, mean | 0.437 | SD 0.031 |  |
|  | usefulness_novice | Intra-rater Cohen’s κ, mean (SD) | 0.794 | SD 0.108 |  |
|  |  | Intra-rater Cohen’s κ, median [IQR] | 0.756 | IQR [0.727–0.822] |  |
|  |  | Inter-rater κ, 1st pass | 0.509 |  |  |
|  |  | Inter-rater κ, 2d pass | 0.503 |  |  |
|  |  | Inter-rater κ, mean | 0.506 | SD 0.004 |  |
| **Reliability -**  **% positive/**  **negative agreement** | usefulness | Intra-rater positive / agreement, mean | 93.6% | SD 5.2% |  |
|  |  | Intra-rater negative agreement, mean | 78.2% | SD 20.2% |  |
|  |  | Inter-rater positive agreement, T1–T2 | 88.1–85.3% |  |  |
|  |  | Inter-rater negative agreement, T1–T2 | 57.5–55.8% |  |  |
|  | usefulness_novice | Intra-rater positive / agreement, mean | 90.2% | SD 5.6% |  |
|  |  | Intra-rater negative agreement, mean | 89.2% | SD 5.5% |  |
|  |  | Inter-rater positive / agreement, T1–T2 | 78.0–76.6% |  |  |
|  |  | Inter-rater negative agreement, T1–T2 | 72.8–73.5% |  |  |
| **Hypothese testing for Construct validity** | IoU – usefulness_prop | Pearson r | 0.735 | 95% CI [0.609 ; 0.825] | p < .001 |
|  | IoU – usefulness_novice_prop | Pearson r | 0.830 | 95% CI [0.743 ; 0.890] | p < .001 |
| **Threshold determination** | usefulness proportion ≥ 0.6 | ROC AUC for IoU | 0.934 | 95% CI [0.881–0.987] |  |
|  |  | Youden threshold for IoU | 0.206 | 95% bootstrap CI [0.140–0.230] | Se 0.807;  Sp 1.000 |
|  |  | ROC AUC for seg_latent | 0.979 | 95% CI [0.949–1.000] |  |
|  |  | Youden threshold for seg_latent | 3.510 | 95% bootstrap CI [3.129–4.273] | Se 0.930;  Sp 0.941 |
|  | Novice usefulness proportion ≥ 0.6 | ROC AUC for IoU | 0.977 | 95% CI [0.948–1.000] |  |
|  |  | Youden threshold for IoU | 0.299 | 95% bootstrap CI [0.242–0.306] | Se 0.872;  Sp 1.000 |
|  |  | ROC AUC for seg_latent | 0.987 | 95% CI [0.965–1.000] |  |
|  |  | Youden threshold for seg_latent | 4.689 | 95% bootstrap CI [4.515–5.176] | Se 0.974;  Sp 0.971 |

#### Table 9 - Step 2 (thresholds)

### Reliability of the binary usefulness anchors

The binary usefulness anchors were reproducible, although less robust than the continuous C-SES.

**For *usefulness*, intra-rater** Cohen’s kappa had a mean of 0.723 and a median of 0.789, indicating generally good test–retest reproducibility but with substantial between-rater variability. Inter-rater kappa was 0.458 at the first pass and 0.415 at the second pass, with a descriptive mean of 0.437. Furthermore, individual kappas ranged from 0.372 to 1.000. This indicates moderate agreement across raters and shows that the binary non-expert usefulness judgement was more heterogeneous than the continuous C-SES score.

One rater showed perfect intra-rater agreement for the binary anchor. Inspection of the T1–T2 contingency table confirmed that both positive and negative categories were used and that all classifications were reproduced identically at the second rating round. This was interpreted as a perfect test–retest reproducibility for that rater.

**For *usefulness_novice***, intra-rater reliability was slightly higher and more homogeneous, with a mean kappa of 0.794 and a median of 0.756. Inter-rater kappa was 0.509 at the first pass and 0.503 at the second pass, with a mean of 0.506. Individual kappas ranged from 0.703 to 1.000. This suggests more balanced and reproducible novice-usefulness judgements.

**Positive and negative agreement** clarified the pattern behind the kappa values. For *usefulness*, intra-rater positive agreement was high at 93.6%, whereas negative agreement was lower and more variable at 78.2%. Inter-rater positive agreement was also higher than negative agreement, with positive agreement of 88.1% and 85.3% at T1 and T2, compared with negative agreement of 57.5% and 55.8%. This suggests that raters more consistently agreed when a segmentation was useful than when it was not useful.

For *usefulness_novice*, agreement was more balanced. Intra-rater positive and negative agreement were 90.2% and 89.2%, respectively. Inter-rater positive agreement was 78.0% and 76.6% at T1 and T2, while negative agreement was 72.8% and 73.5%. These results indicate that novice-usefulness classifications were more symmetrical across positive and negative categories.

### Relationship between IoU and usefulness

The exploratory association between IoU and usefulness proportions was strong. Pearson’s r was 0.735 for non-expert usefulness and 0.830 for novice usefulness. In linear regression, each 0.10 increase in IoU was associated with a 12.2 percentage-point increase in non-expert usefulness and a 17.3 percentage-point increase in novice usefulness. IoU explained 54.1% of the variance in non-expert usefulness and 69.0% of the variance in novice usefulness.

Unsurprisingly, IoU explained more of the variance of usefulness for novices than for non-experts, suggesting that the quality of the segmentation alone matters more for novices to help guide the procedure.

These findings show that objective overlap was strongly related to clinical usefulness, especially for novice-oriented usefulness. However, IoU did not explain the entire variability in usefulness: approximately 45.9% of the variance remained unexplained for non-expert anaesthesiologists and 31.0% for novices. This suggests that perceived clinical usefulness is not determined by segmentation overlap alone, but probably also reflects other factors such as clinical experience, confidence in procedural guidance, and rater-specific clinical judgement.

### Threshold results

The primary threshold analyses used the 0.6 usefulness cut-off. For the non-expert reference, IoU showed high discrimination, with an AUC of 0.934. The Youden threshold was 0.206, with sensitivity of 0.807 and specificity of 1.000. The latent C-SES score showed even higher discrimination, with an AUC of 0.979 and a Youden threshold of 3.510, yielding sensitivity of 0.930 and specificity of 0.941.

For the novice reference, discrimination was also high. IoU had an AUC of 0.977 and a Youden threshold of 0.299, with sensitivity of 0.872 and specificity of 1.000. The latent C-SES score had an AUC of 0.987 and a Youden threshold of 4.689, with sensitivity of 0.974 and specificity of 0.971.

#### Internal validation and full diagnostic reporting

The following results complete the threshold-derivation step at the primary Youden operating point (0.6 cut-off). Positive and negative sequence counts by cut-off are given in Table 10a, confusion matrices with predictive values and 95% bootstrap confidence intervals in Table 10b, and optimism-corrected estimates (Harrell enhanced bootstrap, 2000 resamples) in Table 10c.

| **Usefulness cut-off** | **Non-expert positive** | **Non-expert negative** | **Novice positive** | **Novice negative** |
| --- | --- | --- | --- | --- |
| 0.5 | 62 | 12 | 43 | 31 |
| 0.6 (primary) | 57 | 17 | 39 | 35 |
| 0.7 | 54 | 20 | 32 | 42 |
| 0.8 | 44 | 30 | 25 | 49 |
| 0.9 | 37 | 37 | 20 | 54 |

*Table 10a. Positive/negative sequence counts by usefulness cut-off (n = 74).*

| **Analysis (Youden threshold)** | **n (pos/neg)** | **TP/FP/FN/TN** | **Sensitivity [95% CI]** | **Specificity [95% CI]** | **PPV [95% CI]** | **NPV [95% CI]** | **AUC [95% CI]** |
| --- | --- | --- | --- | --- | --- | --- | --- |
| IoU – non-expert (0.206) | 74 (57/17) | 46/0/11/17 | 0.807 [0.719–0.947] | 1.000 [0.882–1.000] | 1.000 [0.964–1.000] | 0.607 [0.515–0.824] | 0.934 [0.869–0.978] |
| seg_latent – non-expert (3.510) | 74 (57/17) | 53/1/4/16 | 0.930 [0.789–1.000] | 0.941 [0.824–1.000] | 0.981 [0.948–1.000] | 0.800 [0.586–1.000] | 0.979 [0.941–0.999] |
| IoU – novice (0.299) | 74 (39/35) | 34/0/5/35 | 0.872 [0.821–1.000] | 1.000 [0.886–1.000] | 1.000 [0.902–1.000] | 0.875 [0.833–1.000] | 0.977 [0.940–0.999] |
| seg_latent – novice (4.689) | 74 (39/35) | 38/1/1/34 | 0.974 [0.897–1.000] | 0.971 [0.942–1.000] | 0.974 [0.944–1.000] | 0.971 [0.897–1.000] | 0.987 [0.961–1.000] |

*Table 10b. Confusion matrix, sensitivity, specificity, predictive values and area under the ROC curve, with 95% bootstrap confidence intervals, at the Youden operating point (0.6 cut-off). Predictive values are prevalence-dependent (see text).*

| **Analysis** | **AUC** | **Sensitivity** | **Specificity** | **Youden** | **PPV** | **NPV** |
| --- | --- | --- | --- | --- | --- | --- |
| IoU – non-expert | 0.934 → 0.935 | 0.807 → 0.795 | 1.000 → 0.961 | 0.807 → 0.756 | 1.000 → 0.987 | 0.607 → 0.574 |
| seg_latent – non-expert | 0.979 → 0.979 | 0.930 → 0.921 | 0.941 → 0.906 | 0.871 → 0.828 | 0.981 → 0.971 | 0.800 → 0.774 |
| IoU – novice | 0.977 → 0.977 | 0.872 → 0.849 | 1.000 → 0.977 | 0.872 → 0.826 | 1.000 → 0.980 | 0.875 → 0.851 |
| seg_latent – novice | 0.987 → 0.987 | 0.974 → 0.961 | 0.971 → 0.959 | 0.946 → 0.920 | 0.974 → 0.963 | 0.971 → 0.957 |

*Table 10c. Apparent versus optimism-corrected operating characteristics (apparent → corrected).*

Optimism was negligible for AUC (≤0.001), consistent with discrimination based on a single raw metric. Threshold-dependent characteristics showed only modest optimism (corrected Youden index lower by 0.03–0.05). Predictive values depend on the prevalence of this spectrum-enriched sample (77% of sequences positive for non-expert and 53% for novice usefulness at the 0.6 cut-off) and are therefore not clinical predictive values; sensitivity and specificity remain the primary, prevalence-independent characteristics.

The higher thresholds for novice usefulness are clinically intuitive: a segmentation must be of higher quality to be useful for a novice than for a non-expert anaesthesiologist. The C-SES thresholds also show that the subjective scale can be translated into clinically meaningful operating points.

#### Results for alternative cut-offs and thresholds selection rules

Those sensitivity analyses are presented here because they are in alignment with the previous results. They are shown in Figure 7.

#### Figure 7: Thresholds for non-experts anaesthetist (top) and for novices (bottom).

All methods (Youden, specificity first, sensitivity first) and all thresholds (0.5, 0.6, 0.7, 0.8, 0.9) are represented. For clarity, CI 95% is given only for Youden.

*IoU : Intersection over Union; seg_latent: subjective segmentation score (aggregated)*

Threshold analyses showed a consistent increase in the selected operating points when stricter usefulness cut-offs were applied. For the non-expert-oriented reference, Youden thresholds increased from 0.206 to 0.304 for IoU and from 3.51 to 4.83 for the latent C-SES score as the usefulness cut-off increased from 0.5 to 0.9. For the novice-oriented reference, the corresponding thresholds increased from 0.288 to 0.363 for IoU and from 4.34 to 6.42 for the latent C-SES score. This pattern indicates that stricter definitions of clinical usefulness require higher segmentation quality.

The novice-oriented thresholds were consistently higher than the non-expert-oriented thresholds, supporting the clinical interpretation that highlighting must be of higher quality to be useful for novices. Across cut-offs, the latent C-SES score generally showed higher discriminative performance than IoU, suggesting that it captured clinically relevant aspects of perceived segmentation quality beyond geometric overlap alone.

The three threshold-selection rules provided complementary interpretations. The Youden index gave the best overall balance between sensitivity and specificity and was therefore retained as the primary rule. The sensitivity-constrained rule produced more permissive thresholds, appropriate when the priority is to minimise missed useful segmentations. Conversely, the specificity-constrained rule produced more conservative thresholds, appropriate when the priority is to avoid accepting segmentations that are not clinically useful. In several analyses, the constrained rules yielded the same threshold as Youden, indicating that the Youden threshold already satisfied the corresponding 90% operating constraint.

At very strict usefulness cut-offs, especially 0.8 and 0.9, threshold estimates should be interpreted more cautiously. Although discrimination remained generally high, the binary reference became more selective, and the resulting estimates were more dependent on the distribution of cases within the sample. These analyses therefore support robustness of the main findings, but should be considered sensitivity analyses rather than definitive operating points.

# Sensitivity analyses

Table 11 shows a synoptic view of the main sensitivity analysis results for the alternative metric DSC, and region specific-analysis.

|  | **IoU** | **IoU - femoral** | **IoU - brachial** | **IoU - sciatic** | **DSC** | **DSC - femoral** | **DSC - brachial** | **DSC - sciatic** |
| --- | --- | --- | --- | --- | --- | --- | --- | --- |
| **Pearson r** | 0.861***  95% CI [0.787 ; 0.910] | 0.822***  95% CI [0.597 ; 0.927] | 0.895***  95% CI [0.769 ; 0.954] | 0.891***  95% CI [0.781 ; 0.947] | 0.868***  95% CI [0.798 ; 0.915] | 0.799***  95% CI [0.552 ; 0.917] | 0.908***  95% CI [0.797 ; 0.960] | 0.899***  95% CI [0.796 ; 0.951] |
| **Slope β** | 0.075***  95% CI [0.065 ; 0.085] | 0.074***  95% CI [0.049 ; 0.099] | 0.079***  95% CI [0.062 ; 0.097] | 0.076***  95% CI [0.061 ; 0.091] | 0.091***  95% CI [0.079 ; 0.103] | 0.092***  95% CI [0.057 ; 0.126] | 0.096***  95% CI [0.076 ; 0.115] | 0.091***  95% CI [0.074 ; 0.108] |
| **R²** | 0.741 | 0.676 | 0.801 | 0.794 | 0.753 | 0.639 | 0.825 | 0.808 |
| **ICC intra** | 0.905  SD 0.032 | 0.862  SD 0.098 | 0.874  SD 0.066 | 0.927  SD 0.023 | 0.905  SD 0.032 | 0.862  SD 0.098 | 0.874  SD 0.066 | 0.927  SD 0.023 |
| **ICC inter (2,1)** | 0.689  95% CI [0.603 ; 0.770] | 0.516  95% CI [0.329 ; 0.719] | 0.795  95% CI [0.679 ; 0.890] | 0.713  95% CI [0.587 ; 0.827] | 0.689  95% CI [0.603 ; 0.770] | 0.516  95% CI [0.329 ; 0.719] | 0.795  95% CI [0.679 ; 0.890] | 0.713  95% CI [0.587 ; 0.827] |
| **ICC inter (2,3)** | 0.869  95% CI [0.820 ; 0.909] | 0.762  95% CI [0.595 ; 0.885] | 0.921  95% CI [0.864 ; 0.960] | 0.881  95% CI [0.810 ; 0.935] | 0.869  95% CI [0.820 ; 0.909] | 0.762  95% CI [0.595 ; 0.885] | 0.921  95% CI [0.864 ; 0.960] | 0.881  95% CI [0.810 ; 0.935] |
| **ICC inter (2,k)** | 0.940  95% CI [0.914 ; 0.959] | 0.882  95% CI [0.771 ; 0.947] | 0.964  95% CI [0.937 ; 0.983] | 0.946  95% CI [0.908 ; 0.971] | 0.940  95% CI [0.914 ; 0.959] | 0.882  95% CI [0.771 ; 0.947] | 0.964  95% CI [0.937 ; 0.983] | 0.946  95% CI [0.908 ; 0.971] |
| **κ intra usefulness** | 0.723  SD 0.245 | 0.698  SD 0.381 | 0.646  SD 0.292 | 0.789  SD 0.189 | 0.723  SD 0.245 | 0.698  SD 0.381 | 0.646  SD 0.292 | 0.789  SD 0.189 |
| **κ inter usefulness** | 0.437  SD 0.031 | 0.095*^††^*  SD 0.015 | 0.522  SD 0.015 | 0.530  SD 0.092 | 0.437  SD 0.031 | 0.095*^††^*  SD 0.015 | 0.522  SD 0.015 | 0.530  SD 0.092 |
| **κ intra usefulness novice** | 0.794  SD 0.108 | 0.682  SD 0.176 | 0.810  SD 0.132 | 0.857  SD 0.103 | 0.794  SD 0.108 | 0.682  SD 0.176 | 0.810  SD 0.132 | 0.857  SD 0.103 |
| **κ inter usefulness novice** | 0.506  SD 0.004 | 0.383  SD 0.002 | 0.594  SD 0.076 | 0.509  SD 0.050 | 0.506  SD 0.004 | 0.383  SD 0.002 | 0.594  SD 0.076 | 0.509  SD 0.050 |
| **AUC Non-Expert** | 0.934  95% CI [0.881 ; 0.987] | 0.806  95% CI [0.576 ; 1.000] | 0.958  95% CI [0.883 ; 1.000] | 0.983  95% CI [0.949 ; 1.000] | 0.934  95% CI [0.881 ; 0.987] | 0.806  95% CI [0.576 ; 1.000] | 0.958  95% CI [0.883 ; 1.000] | 0.983  95% CI [0.949 ; 1.000] |
| **Threshold Youden Non-Expert** | 0.206  95% CI [0.140 ; 0.230]  Se 0.807; Sp 1.000 | 0.200  95% CI [0.087 ; 0.240]  Se 0.722; Sp 1.000 | 0.230  95% CI [0.167 ; 0.247]  Se 0.882; Sp 1.000 | 0.159  95% CI [0.068 ; 0.209]  Se 0.909; Sp 1.000 | 0.342  95% CI [0.246 ; 0.373]  Se 0.807; Sp 1.000 | 0.333  95% CI [0.160 ; 0.385]  Se 0.722; Sp 1.000 | 0.373  95% CI [0.284 ; 0.394]  Se 0.882; Sp 1.000 | 0.274  95% CI [0.128 ; 0.342]  Se 0.909; Sp 1.000 |
| **AUC Novice** | 0.977  95% CI [0.948 ; 1.000] | 0.939  95% CI [0.832 ; 1.000] | 1.000  95% CI [1.000 ; 1.000] | 0.982  95% CI [0.949 ; 1.000] | 0.977  95% CI [0.948 ; 1.000] | 0.939  95% CI [0.832 ; 1.000] | 1.000  95% CI [1.000 ; 1.000] | 0.982  95% CI [0.949 ; 1.000] |
| **Threshold Youden Novice** | 0.299  95% CI [0.242 ; 0.306]  Se 0.872; Sp 1.000 | 0.294  95% CI [0.228 ; 0.323]  Se 0.818; Sp 1.000 | 0.284  95% CI [0.247 ; 0.316]  Se 1.000; Sp 1.000 | 0.277  95% CI [0.209 ; 0.323]  Se 0.933; Sp 0.933 | 0.460  95% CI [0.389 ; 0.468]  Se 0.872; Sp 1.000 | 0.455  95% CI [0.371 ; 0.487]  Se 0.818; Sp 1.000 | 0.442  95% CI [0.394 ; 0.479]  Se 1.000; Sp 1.000 | 0.433  95% CI [0.342 ; 0.487]  Se 0.933; Sp 0.933 |

#### Table 11 : Main sensitivity analysis.

IoU : Intersection over Union. DSC : Dice Similarity Coefficient. CI : confidence interval. SD : Standard Deviation. IQR : Interquartile Range. ICC : Intraclass Correlation Coefficient. RSE : Residual Standard Error. AUC : Area Under the Curve. *** : p value < 0.001

*†† The low inter-rater kappa values observed in the femoral subgroup reflect extreme class imbalance (e.g. 18 of 20 sequences classified as positive for the usefulness item) rather than poor rater agreement (Feinstein-Cicchetti paradox). These values do not reflect true agreement and are not interpretable in this context. See Limitations in Appendix 3 for further discussion.*

### Alternative objective metric: DSC

Replacing IoU with DSC produced similar results. Overall, the Pearson correlation between C-SES and DSC was 0.868, compared with 0.861 for IoU. The regression model explained 75.3% of DSC variance, compared with 74.1% for IoU. This confirms that the main validity findings were robust to the choice of overlap metric.

The threshold results were also consistent. For non-expert usefulness, the DSC threshold was 0.342, with the same high discriminative performance as the IoU-based analysis. For novice usefulness, the DSC threshold was 0.460. These thresholds correspond well with the IoU thresholds and support the interpretation that relatively modest overlap values may be clinically useful in this specific context.

### Region-specific analyses

Region-specific analyses showed broadly consistent but less stable results, as expected in smaller subgroups.

For the **femoral** region (N = 20), the correlation between C-SES and IoU was 0.822, and the model explained 67.6% of the variance. The DSC correlation was 0.799, with R² of 0.639. Inter-rater reliability was lower in this region, with ICC(2,1) of 0.516, ICC(2,3) of 0.762, and ICC(2,k=7) of 0.882. This suggests that the femoral subset was somewhat less stable, particularly for single-rater scoring.

The low inter-rater kappa values observed in the femoral subgroup reflect extreme class imbalance (e.g. 18 of 20 sequences classified as positive for the usefulness item) rather than poor rater agreement (Feinstein-Cicchetti paradox). These values do not reflect true agreement and are not interpretable in this context. See Limitations in Appendix 3 for further discussion.

For the **brachial** region (N = 24), results were very strong. The correlation with IoU was 0.895, with R² of 0.801, and the correlation with DSC was 0.908, with R² of 0.825. Inter-rater reliability was also high, with ICC(2,1) of 0.795, ICC(2,3) of 0.921, and ICC(2,k=7) of 0.964. This indicates strong consistency of the framework in the brachial region.

For the **sciatic** region (N = 30), the correlation with IoU was 0.891, with R² of 0.794, and the correlation with DSC was 0.899, with R² of 0.808. Inter-rater reliability was also good, with ICC(2,1) of 0.713, ICC(2,3) of 0.881, and ICC(2,k=7) of 0.946. These findings support the robustness of the C-SES across the sciatic region.

Overall, the region-specific analyses support the applicability of the framework across anatomical contexts, while showing that precision is lower in smaller subgroups and that reliability may vary by region.

Region-specific threshold analyses

Threshold analyses were broadly consistent across anatomical regions. For non-expert usefulness, IoU thresholds were 0.200 in the femoral region, 0.230 in the brachial region, and 0.159 in the sciatic region. Corresponding DSC thresholds were 0.333, 0.373, and 0.274. For novice usefulness, IoU thresholds were 0.294, 0.284, and 0.277, and corresponding DSC thresholds were 0.455, 0.442, and 0.433.

These results suggest that novice-oriented thresholds were more consistent across regions, whereas non-expert thresholds showed greater regional variation. This may reflect differences in anatomical difficulty, rater expectations, or the distribution of usefulness classifications within each subgroup.

### Sequence-level versus frame-level aggregation

The primary analysis used sequence-level IoU. Sensitivity analyses using frame-wise IoU averaged at sequence level yielded similar results (Table 13). Pearson’s r was 0.856 for frame-wise averaged IoU, compared with 0.861 for the sequence-level IoU. R² was 0.7333 compared with 0.7405. Threshold analyses also remained broadly consistent. The non-expert IoU threshold shifted from 0.206 to 0.232, and the novice threshold shifted from 0.299 to 0.264.

For DSC, sequence-level and frame-wise averaged analyses were nearly identical for validity, with Pearson’s r of 0.868 in both cases and R² of approximately 0.753. This indicates that the findings were not driven by a single aggregation strategy for the objective metric.

### Sensitivity analysis using the arithmetic mean of ratings

Replacing the mixed-effects-derived latent C-SES score with the arithmetic mean of the 14 raw ratings yielded virtually identical validity results (Pearson r and Spearman rho = 1, p < .001). This indicates that the main association with objective overlap metrics was not dependent on the exact aggregation method. However, the mixed-effects model remains preferable because it explicitly accounts for rater and test–retest variability.

### Sensitivity analysis using latent usefulness probabilities

The proportion-based usefulness aggregation was also compared with latent probabilities derived from a mixed-effects logistic model. The correlation between the observed usefulness proportions and the latent probabilities was extremely high (Pearson r ≈ 1.00, p < .001), indicating that the simpler proportion-based approach was sufficient for the main analyses. This supports the use of observed proportions as transparent and clinically interpretable anchors for threshold derivation.

### Exclusion of overlapping raters: the scale-development rater and the reference annotators

Two sensitivity analyses examined the dependency introduced by raters who also contributed to the study. First, because one rater (BD) contributed substantially to the development of the measurement approach, the analyses were repeated after excluding this rater (six raters). Second, to address the overlap between the reference annotators and the C-SES raters (reviewer point 4), the analyses were repeated after excluding all three annotators (BD, KG and YE), leaving four fully independent raters (BM, KY, TG and TGI). In both cases the results were essentially unchanged (Table 12): concurrent validity remained strong (Pearson r = 0.861 for the full sample, 0.838 with six raters and 0.885 with four independent raters, and Spearman ρ = 0.895 [95% CI 0.839–0.928] with four raters); reliability remained in the same interpretive category; IoU-based thresholds and AUCs were essentially unchanged; and the seg_latent thresholds shifted only modestly. This indicates that neither the involvement of the scale-development rater nor the partial annotator–rater overlap materially affected the findings.

| **Result** | **Full sample**  **(7 raters)** | **Excluding scale-development rater (6 raters)** | **Four independent raters (annotators excluded)** |
| --- | --- | --- | --- |
| **Concurrent validity — Pearson r** | 0.861 | 0.838 | 0.885 |
| **Intra-rater ICC(2,1), mean** | 0.905 | 0.903 | 0.895 |
| **Inter-rater ICC(2,3) / ICC(2,k)** | 0.869 / 0.940 | 0.855 / 0.922 | 0.924 / 0.942 |
| **Youden IoU threshold — non-expert / novice** | 0.206 / 0.299 | 0.206 / 0.299 | 0.188 / 0.288 |
| **Youden seg_latent threshold — non-expert / novice** | 3.510 / 4.689 | 3.921 / 4.883 | 3.236 / 4.270 |
| **AUC (IoU) — non-expert / novice** | 0.934 / 0.977 | 0.934 / 0.977 | 0.930 / 0.947 |

*Table 12. Sensitivity analyses excluding the scale-development rater (six raters) and all three reference annotators (four independent raters): main results.*

### Overall interpretation of supplementary results

Overall, the supplementary results support the robustness of the main findings. The C-SES showed strong association with standard objective overlap metrics, high test–retest reliability, and improved measurement precision when ratings were aggregated across multiple experts. The usefulness anchors were sufficiently reproducible to support exploratory threshold derivation, although their inter-rater agreement was lower than that of the continuous C-SES and varied depending on the balance of positive and negative classifications.

Sensitivity analyses confirmed that the framework was robust across IoU and DSC, across sequence-level and frame-wise aggregation strategies, and across anatomical regions. The main limitations were reduced precision in smaller subgroups and non-estimable ROC results when the binary usefulness reference contained only one class. These findings support the internal robustness of the proposed validation framework, while confirming the need for external validation in larger datasets, additional anatomical contexts, and other segmentation platforms.

# Assumption checks

All assumptions required for statistical testing (described in Appendix 1) were met.

| 95% CI are in [] | **IoU sequence-level** | **IoU frame-level averaged** | **DSC sequence-level** | **DSC frame-level averaged** |
| --- | --- | --- | --- | --- |
| **Step 1** | | | | |
| **Mixed model intercept** | 5.017 | 5.017 | 5.017 | 5.017 |
| **SD(patient)** | 2.104 | 2.104 | 2.104 | 2.104 |
| **SD(evaluator)** | 0.471 | 0.471 | 0.471 | 0.471 |
| **SD(evaluator:retest)** | 0.183 | 0.183 | 0.183 | 0.183 |
| **Residual SD** | 1.374 | 1.374 | 1.374 | 1.374 |
| **Pearson r** | 0.861  [0.787 ; 0.910] | 0.856  [0.781 ; 0.907] | 0.868  [0.798 ; 0.915] | 0.868  [0.798 ; 0.915] |
| **Spearman rho** | 0.866  [0.804 ; 0.904] | 0.856  [0.785 ; 0.896] | 0.872  [0.808 ; 0.908] | 0.862  [0.792 ; 0.902] |
| **Linear regression slope β** | 0.075  [0.065 ; 0.085]  R²=0.7405; RSE=0.0926 | 0.073  [0.063 ; 0.084]  R²=0.7333; RSE=0.0921 | 0.091  [0.079 ; 0.103]  R²=0.7533; RSE=0.1086 | 0.092  [0.079 ; 0.104]  R²=0.7536; RSE=0.1093 |
| **Step 2** | | | | |
| **ROC AUC — metric (Non-Expert)** | 0.934  [0.881 ; 0.987] | 0.921  [0.861 ; 0.980] | 0.934  [0.881 ; 0.987] | 0.915  [0.852 ; 0.978] |
| **Youden threshold — metric (Non-Expert)** | 0.206  [0.140 ; 0.230]  Se 0.807; Sp 1.000 | 0.232  [0.155 ; 0.243]  Se 0.789; Sp 1.000 | 0.342  [0.246 ; 0.373]  Se 0.807; Sp 1.000 | 0.336  [0.193 ; 0.347]  Se 0.789; Sp 1.000 |
| **ROC AUC — seg_latent (Non-Expert)** | 0.979  [0.949 ; 1.000] | 0.979  [0.949 ; 1.000] | 0.979  [0.949 ; 1.000] | 0.979  [0.949 ; 1.000] |
| **Youden threshold — seg_latent (Non-Expert)** | 3.510  [3.129 ; 4.273]  Se 0.930; Sp 0.941 | 3.510  [3.129 ; 4.273]  Se 0.930; Sp 0.941 | 3.510  [3.129 ; 4.273]  Se 0.930; Sp 0.941 | 3.510  [3.129 ; 4.273]  Se 0.930; Sp 0.941 |
| **ROC AUC — metric (Novice)** | 0.977  [0.948 ; 1.000] | 0.974  [0.943 ; 1.000] | 0.977  [0.948 ; 1.000] | 0.977  [0.950 ; 1.000] |
| **Youden threshold — metric (Novice)** | 0.299  [0.242 ; 0.306]  Se 0.872; Sp 1.000 | 0.264  [0.253 ; 0.337]  Se 0.974; Sp 0.914 | 0.460  [0.389 ; 0.468]  Se 0.872; Sp 1.000 | 0.410  [0.344 ; 0.460]  Se 0.897; Sp 0.971 |
| **ROC AUC — seg_latent (Novice)** | 0.987  [0.965 ; 1.000] | 0.987  [0.965 ; 1.000] | 0.987  [0.965 ; 1.000] | 0.987  [0.965 ; 1.000] |
| **Youden threshold — seg_latent (Novice)** | 4.689  [4.515 ; 5.176]  Se 0.974; Sp 0.971 | 4.689  [4.515 ; 5.176]  Se 0.974; Sp 0.971 | 4.689  [4.515 ; 5.176]  Se 0.974; Sp 0.971 | 4.689  [4.515 ; 5.176]  Se 0.974; Sp 0.971 |

#### Table 13 – IoU an DSC calculated at the sequence- and frame-level

The sequence-level was the main method, and at frame-level, then averaged was sensitivity analysis.

IoU : Intersection over Union. DSC : Dice Similarity Coefficient. CI : confidence interval. SD : Standard Deviation. IQR : Interquartile Range. ICC : Intraclass Correlation Coefficient. RSE : Residual Standard Error. AUC : Area Under the Curve. *** : p value < 0.001
