# Supplementary material for: Clinical scale of artificial intelligence-based nerve segmentation on ultrasound: a pilot validation study
Source: Front Med (Lausanne). 2026 Aug 20;13:1879747. doi: 10.3389/fmed.2026.1879747 (PMC13538421; doi:10.3389/fmed.2026.1879747)
Supplement: Supplementary file 3 [file Supplementary_file_3.docx]

Appendix 3 – Limitations

[Data	1](#_Toc235109362)

[COSMIN approach	1](#_Toc235109363)

[Data evaluation	2](#_Toc235109364)

[Results interpretation	2](#_Toc235109365)

[Reliability (step 2 - thresholds determination)	3](#_Toc235109366)

[References	4](#_Toc235109367)

# Data

Video sequence was selected to have a whole range of different qualities, since the goal was to correlate various IoU values with various subjective segmentation values. The sequences have been selected only according to the apparent quality of the segmentation, but other criteria could been taken into account (such as BMI, ASA or age).

# COSMIN approach

This study used a COSMIN-informed framework to structure the development and preliminary assessment of the C-SES. COSMIN was originally developed for patient-reported outcome measures (PROM). Although COSMIN methodology allows for the extension of this process to other outcome measurement instruments, including clinician-reported outcome measures (ClinROM) as in this study, this work should be considered an adaptation of the original framework.

A further limitation is that several COSMIN checklists and methodological quality criteria are primarily intended for formal validation studies, whereas the present work remains at the scale-construction and pilot-validation stage. We deliberately applied these procedures at an early stage to make the process transparent and to demonstrate the expected level of methodological rigour, but this should not be interpreted as definitive validation. In particular, content validity was supported by expert consensus and prior literature, but it was not assessed through a formal focus group or structured Delphi process; this will be required in future work.

The risk of bias inherent to scale development was also specifically considered. Selected components of the COSMIN risk-of-bias checklist were provided, and one additional bias source was examined: one rater involved in the study had also substantially contributed to the development of the measurement approach. This is acceptable during scale construction, but would be inappropriate for a definitive validation study unless handled explicitly. To explore this potential bias, all analyses were repeated after excluding the concerned rater, and this was repeated excluding the 3 raters that were involved in the reference annotation, with similar results, supporting the robustness of the findings (Appendix 2 – Table 12).

The three annotaters of the ground truth were also involved in the subjective evaluation step. To moderate this potential bias, the ground truth was annotated 3 weeks after the second subjective annotation. This correspond to the time interval that is recommended for repeated measurements (> 2 weeks). In a future non-preliminary validation step, ground truth annotation and experts evaluation should be performed by different experts.

# Data evaluation

Some errors were made, especially of 2 types :

- Some false positive were taking for true positive. More explicit warning should be given that this should be the base in the instructions.
- Some users may have been influenced by the squestion of the clinical utility even though it was required to not take it into account. A whole set of visual example (and their correspondance with metrics) should be provided in the instructions stages (and not only 3 descriptive examples with words) to show the complete possibilities of AI performance.

A quality check should also be performed after the subjective evaluation by an independent expert, to check for a broadly linear aspect of the data.

# Results interpretation

The degree of relative precision (SDC ≈ 2) suggests that the scale may appear more appropriate for semi-quantative discrimination between poor, intermediate, and good segmentations than for finer distinctions at the individual-rater level, even if the measurement properties were substantially improved when ratings were aggregated across multiple experts (SDC ≈ 2).

**Recommended number of raters and practical advantage over objective annotation.** Because reliability and measurement precision improved markedly with aggregation (single-rater SDC ≈ 2.1 falling to ≈ 1.2 for three raters, and inter-rater reliability rising from moderate for one rater to good — ICC(2,3) ≈ 0.87 — for three), we recommend aggregating at least three expert raters for practical use of the C-SES (even though literature does not formally recommend this number of raters); a single rater remains acceptable only for coarse, semi-quantitative screening. This does not offset the practical advantage of the C-SES over objective annotation, for two reasons. First, multi-rater aggregation is a general recommendation for reference and subjective measurements rather than a burden specific to the C-SES: robust objective ground-truth annotation itself relies on several expert annotators (our own reference standard was generated by a three-expert panel). Second, even aggregated over three raters, the C-SES remains far less time-consuming than objective annotation, because each rating is a single holistic 0–10 score per sequence (seconds to about a minute), whereas objective annotation requires manual pixel-level delineation of the target across many frames of every sequence. Requiring three raters therefore multiplies a very short task, and the total effort remains substantially lower than frame-by-frame manual segmentation.

# Reliability (step 2 - thresholds determination)

Several limitations regarding reliability of step 2 are also worth mentioning.

**Shared-rater dependency between the index test and the clinical reference standard.** In Step 2, the seg_latent score and the usefulness-based binary reference both originate from the same seven raters judging the same sequences. A rater who perceives high segmentation quality is likely to assign both a high segmentation score and a positive usefulness vote, introducing shared variance that compromises the independence between index test and reference standard, a source of bias that tends to overestimate diagnostic accuracy.[(1,2)](https://www.zotero.org/google-docs/?M0OAIg)

Three considerations mitigate this concern. First, the IoU — computed algorithmically, independently of the panel — provides an unbiased comparator, and its AUC values are themselves high (0.934 and 0.977). Second, segmentation quality and clinical usefulness are related but distinct constructs. Third, the sensitivity analysis excluding rater BD confirmed the robustness of the findings.

Nonetheless, AUC estimates for seg_latent versus usefulness may carry a modest upward bias. A split-panel design, with separate rater groups for each judgement, would eliminate this dependency in future studies.

**The binary usefulness items were not formally validated in terms of content validity**: whilst their face validity was considered acceptable by the expert panel, no structured cognitive interview or Delphi process was conducted to confirm that the items comprehensively and unambiguously capture the intended clinical constructs. The hypothesis testing to construct validity that was carried out in this study was an initial, albeit insufficient, step towards examining the validity of this concept.

**Class imbalance and its effect on inter-rater kappa.** Under the primary 0.6 consensus cut-off, 77% of sequences (57/74) were classified as positive for the *usefulness* item. This degree of prevalence imbalance has a well-documented effect on Cohen's kappa: the theoretical upper bound of kappa is constrained by prevalence according to the Feinstein-Cicchetti paradox, such that a perfectly agreeing set of raters cannot achieve a kappa of 1.0 when prevalence departs substantially from 0.5. In the present case, the maximum kappa attainable at a prevalence of 0.77 is approximately 0.46. The observed inter-rater kappa of 0.44 therefore corresponds to 95% of the theoretical ceiling, indicating that rater agreement was in fact near-maximal relative to what the prevalence structure permits. Interpreting this value against conventional benchmarks (e.g. Landis and Koch) without this correction may lead to an unduly pessimistic appraisal of reliability. By contrast, the *usefulness_novice* item had a near-balanced prevalence of 53%, for which the theoretical kappa ceiling approaches 0.95; the observed kappa of 0.51 for this item therefore reflects genuinely moderate agreement and is directly interpretable against standard benchmarks.

**Class imbalance and precision of specificity estimates.** The observed specificity of 1.00 for the IoU threshold (Youden criterion, *usefulness* reference) is based on only 17 negative sequences globally. Whilst the point estimate is consistent with a high-performing threshold, the 95% exact (Clopper-Pearson) confidence interval for this specificity extends as low as 0.81, reflecting the limited number of true negatives available. This imprecision should be borne in mind when interpreting the practical operating characteristics of the derived threshold. The novice-oriented analysis, which benefited from a more balanced class distribution (35 negative sequences), yields more stable specificity estimates and is less affected by this limitation.

# References

[1.](https://www.zotero.org/google-docs/?cUCzhk)	[Whiting PF, Rutjes AWS, Westwood ME, Mallett S, Deeks JJ, Reitsma JB, Leeflang MMG, Sterne JAC, Bossuyt PMM, QUADAS-2 Group. QUADAS-2: a revised tool for the quality assessment of diagnostic accuracy studies. *Ann Intern Med* (2011) 155:529–536. doi: 10.7326/0003-4819-155-8-201110180-00009](https://www.zotero.org/google-docs/?cUCzhk)

[2.](https://www.zotero.org/google-docs/?cUCzhk)	[Lijmer JG, Mol BW, Heisterkamp S, Bonsel GJ, Prins MH, van der Meulen JHP, Bossuyt PMM. Empirical Evidence of Design-Related Bias in Studies of Diagnostic Tests. *JAMA* (1999) 282:1061–1066. doi: 10.1001/jama.282.11.1061](https://www.zotero.org/google-docs/?cUCzhk)
